# New RoxS sRNA Targets Identified in Bacillus subtilis by Pulsed SILAC

**DOI:** 10.1128/spectrum.00471-23

**Published:** 2023-06-20

**Authors:** Delphine Allouche, Gergana Kostova, Marion Hamon, Christophe H. Marchand, Mathias Caron, Sihem Belhocine, Ninon Christol, Violette Charteau, Ciarán Condon, Sylvain Durand

**Affiliations:** a Expression Génétique Microbienne, CNRS, Université Paris Cité, Institut de Biologie Physico-Chimique, Paris, France; b FR550, CNRS, Plateforme de Protéomique, Institut de Biologie Physico-Chimique, Paris, France; c CNRS, UMR7238, Laboratory of Computational and Quantitative Biology, Sorbonne Université, Institut de Biologie Paris-Seine, Paris, France; Paris-Saclay University

**Keywords:** non-coding RNA, translation, *Bacillus subtilis*, RNA degradation, SILAC

## Abstract

Non-coding RNAs (sRNA) play a key role in controlling gene expression in bacteria, typically by base-pairing with ribosome binding sites to block translation. The modification of ribosome traffic along the mRNA generally affects its stability. However, a few cases have been described in bacteria where sRNAs can affect translation without a major impact on mRNA stability. To identify new sRNA targets in Bacillus subtilis potentially belonging to this class of mRNAs, we used pulsed-SILAC (stable isotope labeling by amino acids in cell culture) to label newly synthesized proteins after short expression of the RoxS sRNA, the best characterized sRNA in this bacterium. RoxS sRNA was previously shown to interfere with the expression of genes involved in central metabolism, permitting control of the NAD+/NADH ratio in B. subtilis. In this study, we confirmed most of the known targets of RoxS, showing the efficiency of the method. We further expanded the number of mRNA targets encoding enzymes of the TCA cycle and identified new targets. One of these is YcsA, a tartrate dehydrogenase that uses NAD+ as co-factor, in excellent agreement with the proposed role of RoxS in management of NAD+/NADH ratio in Firmicutes.

**IMPORTANCE** Non-coding RNAs (sRNA) play an important role in bacterial adaptation and virulence. The identification of the most complete set of targets for these regulatory RNAs is key to fully identifying the perimeter of its function(s). Most sRNAs modify both the translation (directly) and mRNA stability (indirectly) of their targets. However, sRNAs can also influence the translation efficiency of the target primarily, with little or no impact on mRNA stability. The characterization of these targets is challenging. We describe here the application of the pulsed SILAC method to identify such targets and obtain the most complete list of targets for a defined sRNA.

## INTRODUCTION

The importance of *trans-*acting small regulatory RNAs (sRNA) in post-transcriptional regulation has been widely demonstrated in bacteria. The early characterization of sRNAs was mostly done in Escherichia coli and its pathogenic relatives. However, in the past decade, an increasing number of sRNAs have also been characterized in Gram-positive bacteria, such as Bacillus subtilis and its virulent kin. These studies have highlighted common features and interesting differences. For example, most sRNAs in Gram-negative bacteria characterized thus far require either the Sm-like protein Hfq or the RNA chaperone ProQ to efficiently bind to their targets (reviewed in reference 1). However, ProQ is absent from Gram-positive bacteria and, although Hfq is present, this protein is not generally required for efficient base-pairing and regulation of most sRNAs characterized so far, with relatively few exceptions (2–4).

In most cases, sRNAs bind at or close to the ribosome binding site (RBS) and inhibit mRNA translation. This often indirectly results in mRNA destabilization because ribosomes no longer protect the transcript from attack by ribonucleases (RNases). Small RNAs can also bind outside of the RBS and directly provoke mRNA degradation, by changing RNA structure or by directly recruiting RNases (5, 6). sRNAs can also positively regulate the expression of some targets by modifying the secondary structure of the 5′ untranslated region (5′-UTR) to release the RBS and increase translation efficiency, or change structure to impair RNase cleavages (reviewed in reference 7).

Very few cases have been described in bacteria where sRNAs act at the translational level without affecting mRNA stability (8). A recent study in Staphylococcus aureus identified two targets of the IsrR RNA, induced during iron starvation, that are primarily affected at the translational level (9). In all such cases studied thus far, the sRNA base-pairs with the RBS to inhibit translation.

The favored technique for global study of the effects of sRNAs on translation has been to compare proteomes in wild-type (WT) *versus* a strain deleted for the sRNA of interest. The main drawback of this approach is that it only provides a picture of the proteome at equilibrium, with numerous potential indirect effects. This issue has been partially circumvented by inducing sRNA expression for short periods of time (5 to 15 min induction), allowing direct or indirect repressive effects on mRNA levels to be detected due to their short half-lives. However, the half-lives of proteins can be much longer than these induction times, making downregulation of translation much more difficult to measure and potentially missed entirely in cases where the indirect effect on mRNA stability is minimal.

In this work, we have combined RNA sequencing (RNAseq) with pulsed SILAC (stable isotope labeling by amino acids in cell culture), first used in eukaryotes to identify miRNA targets (10), with a goal of identifying new sRNA targets in B. subtilis that potentially include some targets with primarily translational effects. To benchmark the approach, we chose to overexpress the RoxS sRNA, one of the best characterized sRNAs in Gram-positive bacteria, for a short period of time. RoxS is conserved in both B. subtilis and S. aureus, and uses three single-stranded C-rich regions (CRR1-3) to regulate the expression of many genes involved in central metabolism. In B. subtilis, its main proposed function is to adjust temporary imbalances in the NAD+ to NADH ratio, by controlling key steps in fermentation pathways and the TCA cycle. Several direct targets of this sRNA have already been identified by the methods described above, allowing us to validate the performance of the pulsed SILAC experiment. In addition to identifying most of the known RoxS targets, we discovered several new mRNAs potentially regulated by this sRNA. Indeed, our data suggests that RoxS downregulates the expression of all but one enzyme of the TCA cycle, a far greater impact than previously appreciated. As anticipated, we also identified some potential targets that were primarily regulated at the translational level, with only minor effects on mRNA stability. The potential repercussions of this type of regulation are discussed.

## RESULTS

Pulsed SILAC consists of stable isotopic labeling by amino acids of newly synthetized proteins to allow their detection and quantification by mass spectrometry (MS). We performed experiments in a lysine auxotrophic strain to maximize the efficiency of lysine isotope incorporation into new proteins. The strain lacked the native version of the *roxS* gene and expressed RoxS under the control of an arabinose-inducible promoter from the *amyE* locus. The control strain carried the same arabinose promoter inserted at the *amyE* locus with the RoxS sRNA replaced by a transcriptional terminator to avoid polar effects on downstream genes. To ensure appropriate expression of mRNA targets, both strains were cultivated in controlled medium (MD) containing malate, a carbon source we have previously shown strongly induces RoxS expression (11). All 20 amino acids in their light isotopic form were supplied to the medium, but lysine was limited to an empirically determined concentration that allowed it to be almost completely consumed by mid-exponential phase (OD_600_ = 0.6), leading to a growth arrest unless further supplemented with lysine isotopes (Fig. S1). At this point, arabinose and medium-heavy lysine were added to the control strain, and arabinose and heavy lysine were added to the RoxS overexpressing strain, to simultaneously induce RoxS expression and label newly synthesized proteins. After 15-min induction, bacteria were harvested to extract total RNA and soluble proteins. RNA was used for RNAseq analysis and equal amounts of extracted proteins were mixed and subjected to quantitative proteomic analysis. MS-based quantitative data (medium to light, heavy to light, and medium to heavy ratios) determined at the peptide level for each lysine-containing peptide were further used to relatively quantify newly synthesized proteins in both conditions and determine those that were differently expressed upon RoxS induction.

**FIG 1:**
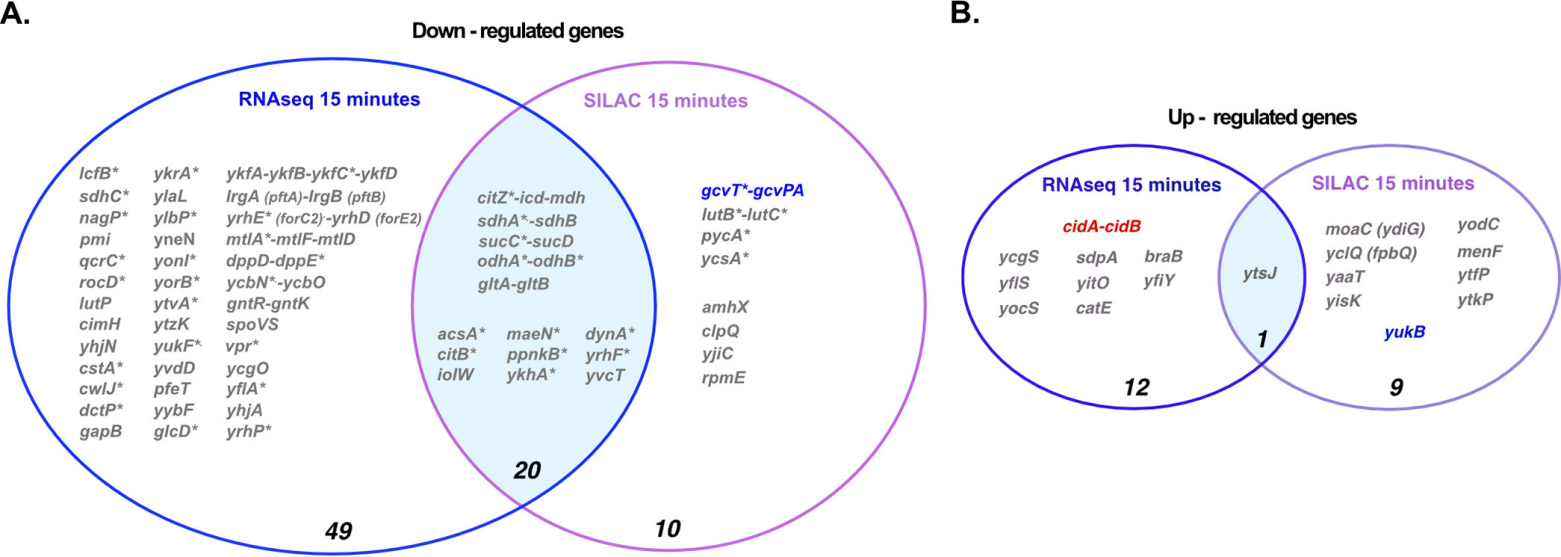
Venn diagrams of genes whose expression is altered at mRNA and protein levels after 15 min of RoxS overexpression. (A) Downregulated genes. (B) Upregulated genes. The number of genes identified by RNAseq, SILAC, or both, is indicated. (*) Indicates genes with a predicted or demonstrated binding site for RoxS in the Shine-Dalgarno sequences. Candidates where no base-pairing could be predicted between the mRNA and RoxS by IntaRNA are shown in red (see Materials and Methods). mRNAs shown in blue were present in the RNAseq data set, but the fold change was just below the threshold. These mRNAs thus potentially belong in the intersection of the Venn diagram.

The up- and downregulated genes identified by RNAseq and by MS analysis after 15 min induction of RoxS are presented in Fig. 1 and Tables 1 and 2. Only statistically significant alterations in expression (*P*-value ≤0.05) with a fold change <0.7 and >1.4 for negatively and positively regulated targets respectively were selected. This threshold includes all previously confirmed targets of RoxS in B. subtilis. As developed below, most of the new potential targets identified fit perfectly well with the proposed function of RoxS in the regulation of the NAD+/NADH ratio via the modulation of carbon metabolism (11–13).

**TABLE 1:** Up- and downregulated genes identified by RNAseq after 15 min induction of RoxS^*a*^

Gene	Protein names	Norm.pXsat15-r1	Norm.pXsat15-r2	Norm.pXsat15-r3	Norm.RoxSt15-r1	Norm.RoxSt15-r2	Norm.RoxS-t15-r3	avg pXsa-t15	avg RoxS-t15	avg ratio RoxS/pXsa
*ppnKB (nadK2)*	NAD kinase 2	6510	5970	6827	568	519	629	6436	572	0.09
*acsA*	Acetyl-coenzyme A synthetase	2565	1941	1571	532	365	198	2026	365	0.17
*sucC*	Succinyl-CoA ligase [ADP-forming] subunit beta	42561	42065	30833	13312	11368	8800	38486	11160	0.29
*ytzK*	Unknown	159	107	110	41	42	26	125	36	0.29
*citZ*	Citrate synthase 2	50651	49889	53625	17845	15011	15705	51388	16187	0.32
*sucD*	Succinyl-CoA ligase [ADP-forming] subunit alpha	30943	30784	23872	10584	9243	7363	28533	9063	0.32
*vpr*	Minor extracellular serine protease	862	692	888	311	253	237	814	267	0.33
*dctP*	Uptake of succinate, fumurate, malate and oxaloacetate via proton symport	6545	5644	3893	2360	1809	1318	5361	1829	0.34
*sdhC*	Succinate dehydrogenase (cytochrome b558 subunit)	3640	3292	2607	1352	1240	943	3180	1178	0.37
*ylbP*	Putative N-acetyltransferase	275	245	278	100	81	119	266	100	0.38
*cimH*	Transporter for citrate (proton symport)	673	633	765	232	274	276	690	261	0.38
*maeN*	Na(+)-malate symporter	8244	7045	8887	3583	2898	3692	8059	3391	0.42
*yrhF*	Unknown	363	319	342	140	144	147	341	144	0.42
*gltB*	Glutamate synthase [NADPH] small chain	22795	18147	15957	9865	9697	5758	18966	8440	0.44
*gltA*	Glutamate synthase, controls GudB activity	49770	39186	38028	21154	21280	14126	42328	18853	0.44
*gapB*	Glyceraldehyde-3-phosphate dehydrogenase, NADP-dependent, gluconeogenic enzyme	10845	5795	6058	4285	2449	3314	7566	3349	0.45
*lrgA (pftA)*	pyruvate transporter	1260	983	930	504	445	485	1058	478	0.45
*ykrA*	Putative HAD superfamily phosphatase	757	682	629	322	372	301	689	332	0.48
*sdhA*	Succinate dehydrogenase flavoprotein subunit	18638	16946	13617	8702	7796	7351	16400	7950	0.49
*pftB (ysbB)*	pyruvate transporter	3117	2341	2150	1269	1265	1183	2536	1239	0.49
*yybF*	Similar to antibiotic resistance protein	838	767	839	399	409	457	815	422	0.52
*yflA*	General stress protein, similar to amino acid carrier protein	268	170	296	130	99	151	245	127	0.52
*yhjN*	Unknown	3499	2904	3646	1894	1687	1613	3350	1731	0.52
*yorB*	Unknown	677	776	639	370	305	415	697	363	0.52
*lutP (yvfH)*	lactate permease	6381	7171	8001	3750	3507	3936	7184	3731	0.52
*citB*	Aconitate hydratase A	157132	138512	128999	80865	75698	64855	141548	73806	0.52
*ykfA*	Similar to immunity to bacteriotoxins	226	221	237	156	131	83	228	123	0.53
*yvcT*	Similar to hydroxypyruvate reductase	2680	2410	2321	1471	1285	1192	2470	1316	0.53
*yrhP*	Similar to efflux protein	246	201	364	163	112	150	270	142	0.53
*yhjA*	Unknown	2548	2737	2342	1322	1449	1310	2542	1360	0.54
*pmi (yvyI)*	Mannose-6-phosphate isomerase	853	734	565	462	415	301	717	393	0.55
*ytvA*	Blue light sensor	1164	1051	1295	651	628	716	1170	665	0.57
*ykfC*	D-glutamyl-L-amino acid peptidase	276	183	297	194	154	92	252	147	0.57
*dppE*	Dipeptide ABC transporter (dipeptide-binding protein)	596	592	747	494	377	259	645	377	0.58
*ykfD*	Similar to oligopeptide ABC transporter (permease)	357	273	387	301	188	116	339	202	0.58
*sdhB*	Succinate dehydrogenase iron-sulfur subunit	10188	9206	8395	5430	5240	5458	9263	5376	0.58
*yneN*	Similar to thiol:disulfide oxidoreductase	5907	5169	4848	3299	3061	2922	5308	3094	0.58
*dppD*	Dipeptide ABC transporter (ATP-binding protein)	226	221	229	155	151	95	225	134	0.59
*ykhA*	Similar to acyl-CoA thioesterase	1108	1136	1333	674	693	746	1192	704	0.59
*cwlJ*	Spore coat protein, cell wall hydrolase	158	92	149	102	65	68	133	78	0.59
*dynA (ypbR)*	Dynamin-like protein, mediates membrane fusion	2693	2570	2360	1605	1607	1376	2541	1529	0.60
*lcfB*	Long-chain fatty-acid-CoA ligase, involved in surfactin production	209	201	167	121	111	115	192	116	0.61
*ykfB*	L-Ala-D/L-Glu epimerase	230	202	259	196	135	99	230	143	0.61
*odhB*	2-oxoglutarate dehydrogenase complex (dihydrolipoamide transsuccinylase, E2 subunit)	23673	22198	21761	13208	12875	15122	22544	13735	0.61
*odhA*	2-oxoglutarate dehydrogenase E1 component	44411	40806	40673	25059	24906	26934	41963	25633	0.61
*iolW (yvaA)*	Scyllo-inositol dehydrogenase, general stress protein	2456	1726	2887	1563	1207	1586	2356	1452	0.62
*ylaL*	Unknown	1806	1801	1910	1201	1086	1135	1839	1141	0.62
*nagP*	N-acetylglucosamine-specific phosphotransferase system, EIICB of the PTS	2029	2057	1762	1256	1295	1084	1949	1212	0.62
*ycbO*	Unknown	1237	693	1184	822	541	548	1038	637	0.62
*ycbN*	Similar to ABC transporter (ATP-binding protein)	1715	996	1487	1101	756	740	1399	866	0.62
*yonI*	Unknown	154	196	202	145	79	132	184	119	0.63
*gntR*	transcriptional repressor (GntR family)	232	141	217	170	80	129	197	126	0.63
*spoVS*	Required for dehydration of the spore core and assembly of the coat	962	819	678	542	541	483	820	522	0.64
*rocD*	Ornithine transaminase	496	411	498	286	309	318	468	304	0.65
*adeR (yukF)*	Transcription activator (PucR family)	877	857	997	638	566	584	910	596	0.66
*putP (ycgO)*	High affinity proline permease	7861	7423	7814	5678	5012	4507	7699	5066	0.66
*yvdD*	Putative lysine decarboxylase	1650	1458	2024	1388	1052	995	1711	1145	0.68
*mtlF*	Mannitol-specific permease of the phosphotransferase system, EIIA of the PTS	150	95	139	97	77	85	128	86	0.68
*icd*	Isocitrate dehydrogenase [NADP]	85972	84535	80312	60308	55680	53564	83606	56517	0.68
*mtlD*	Mannitol-1-phosphate 5-dehydrogenase	338	226	311	218	165	209	292	197	0.68
*mtlA*	Mannitol-specific permease of the phosphotransferase system, EIICB of the PTS, Trigger enzyme	338	198	305	207	174	187	280	189	0.68
*forC2 (yrhE)*	Formate:menadione oxidoreductase	3147	2668	3122	2076	2025	2018	2979	2040	0.69
*forE2 (yrhD)*	Partner subunit of formate:menadione oxidoreductase	813	596	763	479	486	520	724	495	0.69
*glcD*	Probably glycolate oxidase subunit, FAD-binding	429	378	328	274	279	231	378	261	0.69
*mdh*	Malate dehydrogenase	69739	68706	63955	50453	47191	42959	67467	46868	0.69
*cstA*	Similar to pyruvate transporter	370	333	319	256	232	223	341	237	0.70
*zosA (pfeT)*	Fe_2+_ efflux pump, P_1B4_-type ATPase, protects the cell against iron intoxication	587	420	621	438	349	353	543	380	0.70
*qcrC*	Menaquinol:cytochrome c oxidoreductase (cytochrome b/c subunit)	1026	1020	746	709	659	584	931	651	0.70
*gntK*	Gluconokinase	680	418	525	461	310	372	541	381	0.71
*ycgS*	Similar to aromatic hydrocarbon catabolism	121	169	164	206	216	223	151	215	1.42
*yfiY*	ABC transporter for the siderophore schizokinen and arthrobactin (binding protein)	1859	2338	1723	2305	2928	3192	1973	2808	1.43
*dnaE*	Lagging strand DNA polymerase III (alpha subunit), part of the replisome	3903	3913	3825	5214	5663	5851	3880	5576	1.43
*sdpA*	Required for SdpC toxin maturation	214	264	318	426	357	355	265	379	1.44
*catE*	Catechol 2,3-dioxygenase	106	149	148	170	204	209	134	194	1.45
*ytsJ*	Bifunctional malic/malolactic enzyme	5857	6600	7376	8968	9277	10482	6611	9576	1.45
*yocS*	Similar to sodium-dependent bile acid transporter	767	1686	1394	1254	2077	2341	1282	1891	1.48
*cidA (ywbH)*	Holin-like protein	379	386	419	515	589	750	395	618	1.56
*braB*	Branched-chain amino acid transporter	1451	2169	1900	2065	2845	3850	1840	2920	1.57
*ywbG*	Holin-like auxiliary protein	862	983	1221	1493	1544	1795	1022	1611	1.58
*yflS*	Malate transporter	2094	1931	2075	3359	3596	3565	2033	3507	1.73
*yitO*	Unknown	52	80	64	95	122	134	65	117	1.79

a Only statistically significant alterations in expression (*P*-value ≤0.05) with a fold change <0.7 and >1.4 for negatively and positively regulated targets respectively are presented.

**TABLE 2:** Up- and downregulated genes identified by MS analysis after 15 min induction of RoxS^*a*^

Gene	Protein names	Ratio H/M normalized 15min_rep1	Ratio H/M normalized 15min_rep2	Ratio H/M normalized 15min_rep3	Avg ratio	H/M std deviation
*acsA*	Acetyl-coenzyme A synthetase	0.16	NaN	0.10	0.13	0.04
*ppnkB (nadK2)*	NAD kinase 2	0.14	0.15	NaN	0.14	0.01
*citZ*	Citrate synthase 2	0.20	0.18	0.14	0.18	0.03
*yjiC*	Uncharacterized UDP-glucosyltransferase YjiC	0.50	NaN	0.29	0.40	0.15
*gltB*	Glutamate synthase [NADPH] small chain	0.42	0.50	0.32	0.41	0.09
*yrhF*	Uncharacterized protein YrhF	0.44	0.39	NaN	0.42	0.03
*sucC*	Succinyl-CoA ligase [ADP-forming] subunit beta	0.46	0.45	0.44	0.45	0.01
*sucD*	Succinyl-CoA ligase [ADP-forming] subunit alpha	0.52	0.46	0.43	0.47	0.05
*ypbR*	Uncharacterized protein YpbR	0.52	0.46	0.38	0.45	0.07
*gltA*	Glutamate synthase [NADPH] large chain	0.47	0.55	0.34	0.45	0.11
*rpmE*	50S ribosomal protein L31	0.44	0.48	0.53	0.48	0.04
*citB*	Aconitate hydratase A	0.48	0.52	0.49	0.50	0.02
*odhB*	Dihydrolipoyllysine-residue succinyltransferase component of 2-oxoglutarate dehydrogenase complex	0.51	0.52	0.49	0.51	0.02
*odhA*	2-oxoglutarate dehydrogenase E1 component	0.46	0.51	0.63	0.53	0.09
*lutB*	Lactate utilization protein B	0.58	NaN	0.54	0.56	0.03
*clpQ*	ATP-dependent protease subunit ClpQ	0.53	NaN	0.61	0.57	0.06
*amhX*	Amidohydrolase AmhX	0.90	0.61	0.23	0.58	0.33
*yvcT*	Probable 2-ketogluconate reductase	0.61	0.63	0.53	0.59	0.06
*pyc*	Pyruvate carboxylase	0.69	0.61	0.48	0.59	0.11
*lutA*	Lactate utilization protein A	0.73	0.64	0.42	0.60	0.16
*sdhA*	Succinate dehydrogenase flavoprotein subunit	0.58	0.58	0.65	0.61	0.04
*maeN*	Na(+)-malate symporter	0.60	0.67	0.58	0.62	0.05
*ycsA*	Probable tartrate dehydrogenase/decarboxylase	0.47	0.61	0.81	0.63	0.17
*iolW*	scyllo-inositol 2-dehydrogenase (NADP(+))	0.71	0.64	0.57	0.64	0.07
*gcvT*	Aminomethyltransferase	0.74	0.68	0.53	0.65	0.11
*lutC*	Lactate utilization protein C	0.68	0.71	0.60	0.66	0.06
*ykhA*	Uncharacterized acyl-CoA thioester hydrolase YkhA	0.65	NaN	0.68	0.67	0.02
*mdh*	Malate dehydrogenase	0.71	0.70	0.63	0.68	0.05
*icd*	Isocitrate dehydrogenase [NADP]	0.73	0.67	0.66	0.68	0.04
*sdhB*	Succinate dehydrogenase iron-sulfur subunit	0.64	0.78	0.63	0.68	0.09
*ycsF*	UPF0271 protein YcsF	0.99	0.72	0.34	0.68	0.33
*gcvPA*	Probable glycine dehydrogenase (decarboxylating) subunit 1	0.69	1.00	0.69	0.79	0.18
*yodC*	Putative NAD(P)H nitroreductase YodC	1.72	1.08	1.47	1.42	0.32
*ytsJ*	Probable NAD-dependent malic enzyme 4	1.60	1.59	1.36	1.51	0.14
*yisK*	Modulator of Mbl activity, similar to 5-oxo-1,2,5-tricarboxilic-3-penten acid decarboxylase	1.62	1.40	1.79	1.60	0.19
*yukB*	Ftsk domain-containing protein YukB	1.17	1.78	1.91	1.62	0.39
*yaaT*	Stage 0 sporulation protein YaaT	NaN	1.64	1.61	1.62	0.02
*yclQ*	Uncharacterized ABC transporter solute-binding protein YclQ	1.54	1.04	2.54	1.71	0.77
*ytkP*	Probable cysteine synthase	1.68	2.41	NaN	2.04	0.52
*menF*	Isochorismate synthase MenF	3.61	1.04	1.48	2.04	1.37
*moaC*	Cyclic pyranopterin monophosphate synthase accessory protein	4.19	NaN	1.75	2.97	1.73
*ytfP*	putative NAD(FAD)-utilizing dehydrogenase	4.44	2.75	9.04	5.41	3.25

a Only statistically significant alterations in expression (*P*-value ≤0.05) with a fold change <0.7 and >1.4 for negatively and positively regulated targets respectively are presented.

### Genes whose expression is downregulated by RoxS.

**FIG 2:**
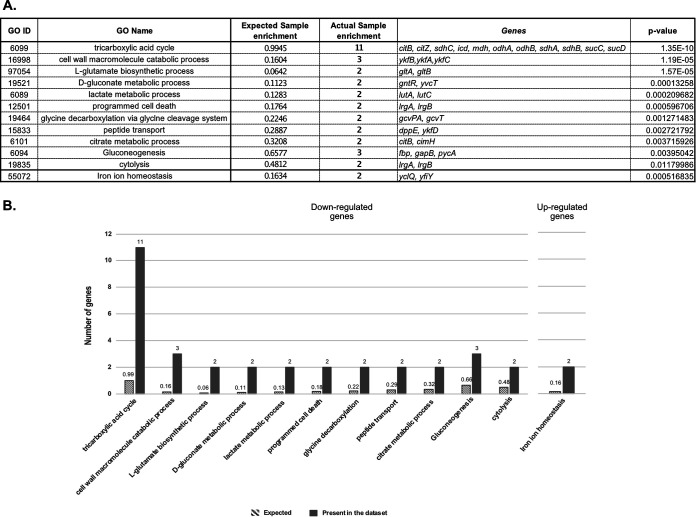
Gene ontology (GO) analysis of genes whose expression is altered at mRNA and protein levels after 15 min of RoxS overexpression. Analysis was performed using the Comparative GO web application (14). (A) Table presenting the different biological functions over-represented in the data set of potential RoxS targets with a *P*-value ≤0.05 (B) Histogram representing the number of genes belonging to each biological function that is over-represented in the downregulated (left graph) and upregulated (right graph) data sets.

**FIG 3:**
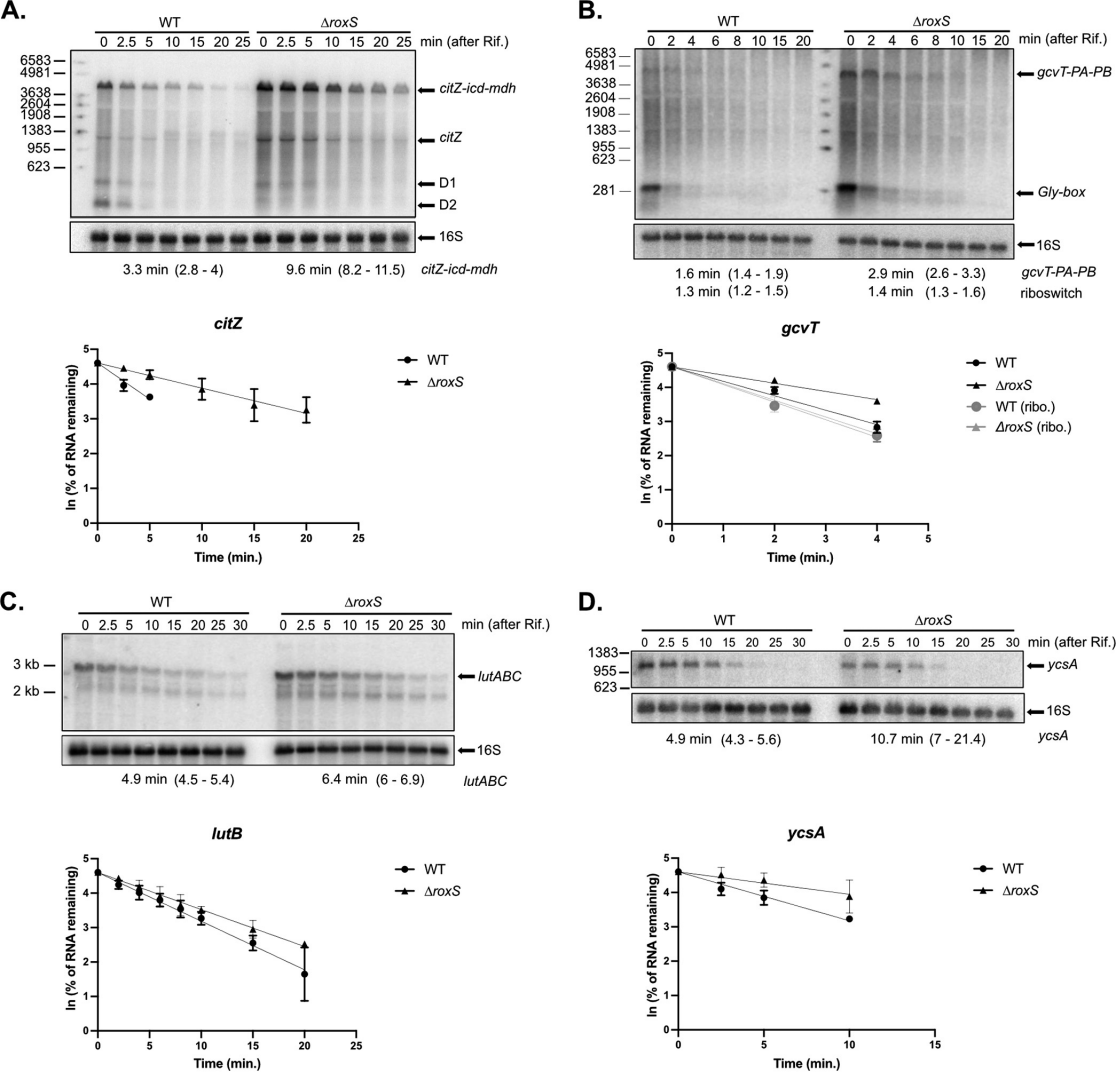
Half-life measurements of candidate RoxS target mRNAs. Northern blot of total RNA isolated from WT and *ΔroxS* mutant strain at different times after addition of rifampicin. Cells were grown in MD + malate (0.5%) and probed for (A) *citZ*, (B) *gcvT*, (C) *lutB*, or in LB + malate (0.5%), and probed for (D) *ycsA.* D1 and D2: degradation products. Northern blots were re-probed for 16S rRNA as a loading control. Graphs and calculated half-lives from two independent experiments are shown beneath the autoradiographs. 95% confidence intervals (CI) are indicated between brackets.

In most cases described in literature, bacterial sRNAs that affect translation also indirectly alter mRNA stability. In agreement with this, we identified 21 potential targets (20 downregulated and one upregulated) that were found in both the RNAseq and the pulsed SILAC data sets (Fig. 1). Among these candidates were numerous previously identified targets of RoxS, such as *ppnkB* (an inorganic polyphosphate/ATP-NAD kinase), *ykhA* (a putative acyl-coenzyme A thioesterase), *acsA*, (an acetyl-CoA synthetase), and the gene encoding the TCA enzyme succinate dehydrogenase, *sucCD* (9–11). Interestingly, we identified eight additional genes encoding enzymes of the TCA cycle, present on four different mRNAs (*citZ*-*icd-mdh*, *citB*, *odhAB*, *sdhAB*). All of these potential targets have at least one predicted base-pairing site for RoxS on the mRNA (Fig. S2). A gene ontology (GO) analysis of the data confirmed the over-representation of the TCA cycle genes (*P*-value of 1 × 10^−10^), compared to other cellular functions (Fig. 2) (14). These results are in agreement with our previous data suggesting that RoxS regulates cell metabolism in order to limit NADH production (13). To confirm a representative example of these TCA cycle targets at the level of mRNA stability, we measured the half-life of the *citZ-icd-mdh* mRNA in a WT and *ΔroxS* mutant strain (Fig. 3A). Two major species of the *citZ* mRNA were detected by Northern blotting. The larger (~4kb) band of corresponds to the tri-cistronic *citZ-icd-mdh* mRNA and the lower band (~1kb) to the mono-cistronic *citZ* transcript. Two degradation products were also visible (D1 and D2), with D2 disappearing in the *ΔroxS* background, suggesting it is stabilized by the presence of RoxS (Fig. 3A). The half-life of *citZ-icd-mdh* mRNA increased 3-fold in a *ΔroxS* strain (3.3 min in a WT strain *vs* 9.6 min in a *ΔroxS* mutant) (Fig. 3A). This result is in good agreement with the effect of RoxS measured by RNAseq (3-fold) (Table 1), but less than the effect detected by SILAC, where the level of the CitZ protein was reduced 5-fold upon over-expression of RoxS (Table 2), suggesting that translational control has a greater impact for this mRNA.

The glycine decarboxylation pathway was also over-represented (*P*-value of 1.2 × 10^−6^) in the GO analysis of the potential target genes identified by pulsed SILAC (Fig. 2). The level of the GcvT protein was reduced 1.5-fold after RoxS induction and the *gcvT-PA-PB* mRNA was reduced 1.3-fold, just below the threshold. The levels of second protein of the operon, GcvPA, were also reduced 1.3-fold. Several putative base-pairing sites for RoxS can be predicted on the *gcvT-PA-PB* mRNA, including in the SD regions of *gcvT* (Fig. S2) and *gcvPB*, suggesting the operon is a bona fide target despite the weak effects. The stability of this mRNA increased 1.8-fold in the absence of RoxS (1.6 min in a WT strain versus 2.9 min in a *ΔroxS* strain; Fig. 3B). Thus, this operon belongs to the category of targets where the effect on translation likely has a similar-magnitude indirect effect on mRNA stability, despite its position in the Venn diagram (Fig. 1). Interestingly, the steady-state level of an ~300-nt transcript corresponding to the glycine riboswitch was also increased in the *ΔroxS* strain. However, the half-life (~1.3 min) was similar in both strains suggesting that RoxS may have an additional indirect impact on the transcription of this operon.

**FIG 4:**
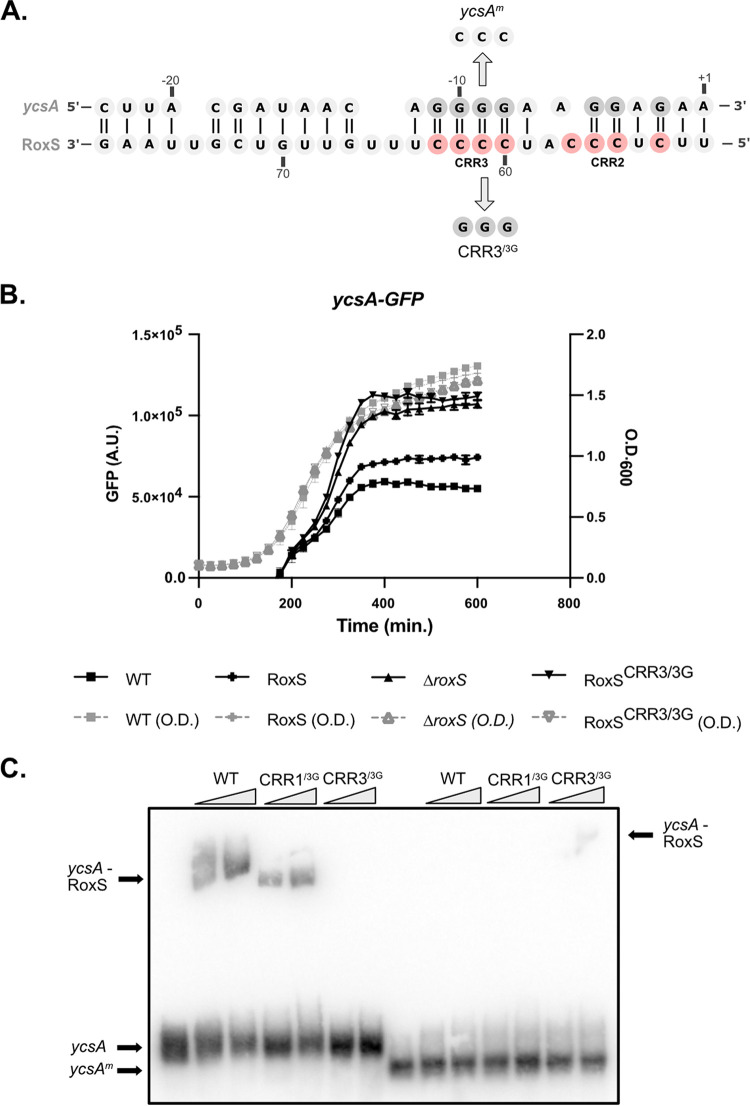
(A) Predicted base-pairing between the SD sequence of *ycsA* and C-rich region (CRR) 2 and 3 of RoxS is shown below the autoradiogram. Variants of RoxS and *ytsJ* mRNA used for EMSA experiment (C) are also indicated. RoxS binds to the *ycsA* Shine-Dalgarno region to regulate translation. (B) Measurement of GFP fluorescence produced by the *ycsA*-GFP translational fusion during growth in LB + malate (0.5%), in WT, *ΔroxS*, RoxS+, and RoxS^CRR3/3G^ cells. For each strain, the rate of fluorescence accumulation was calculated during exponential phase of growth (Fig. S5A) (C) EMSA experiment between the RoxS sRNA (5 and 10 pmol) and the 5′ UTR of the *ycsA* mRNA (5 pmol). The binding of RoxS mutated in CRR1 and/or CRR3 (RoxS^CRR1/3G^: residues 28 to 30, RoxS^CRR3/3G^: residues 60 to 62) was also tested.

Among the downregulated targets that were only identified by pulsed SILAC (and thus potentially regulated primarily at the translational level) were *lutB* and *lutC* of the *lutABC* operon, *ycsA* and *pycA*, all with prospective binding sites for RoxS in their SD regions (Fig. S3; Fig. 4), and four genes (*amhX*, *clpQ*, *rpmE*, and *yjiC*), all with potential binding sites for RoxS in their ORFs (Fig. S4). In the following paragraphs, we will describe the verification of the weak effect of RoxS on the half-lives of two of these potential targets, the *lutABC* and *ycsA* mRNAs, and additional experiments performed on *ycsA* to validate the effect on translation and the predicted RoxS binding site.

The *lutABC* operon encodes lactate utilization enzymes, and interestingly is also regulated in B. subtilis by FsrA, an iron-induced sRNA with similar single-stranded C-rich motifs to RoxS involved in the binding of G-rich Shine-Dalgarno (SD) sequences (15). While FsrA was proposed to base-pair with the SD sequence of *lutA* (15), IntaRNA (16) predicts that both RoxS and FsrA could potentially bind close to the ribosome binding site of each of the three genes of this operon (Fig. S3). The levels of the LutA, LutB, and LutC proteins were downregulated 1.7-fold, 1.8-fold, and 1.5-fold respectively upon RoxS overexpression in the pulsed SILAC experiment. The effect of RoxS on mRNA stability was tested by measuring the impact of the RoxS deletion on the half-life of the *lutABC* mRNA by Northern blotting (Fig. 3C). In agreement with the RNAseq data, its half-life was only weakly affected by the deletion of RoxS (4.9 min in a WT strain versus 6.4 min a *ΔroxS* strain; Fig. 3B), suggesting the effect of RoxS occurs primarily at the translational level.

The regulation of the *ycsA* gene, encoding a putative NAD-dependent tartrate dehydrogenase fits well with the proposed function of RoxS in managing NAD+/NADH ratios. The YcsA protein was reduced 1.6-fold upon RoxS induction in the pulsed SILAC experiment in MD medium. To obtain additional evidence for the translational regulation of *ycsA* by RoxS, we fused the *ycsA* ORF in frame to GFP and determined its rate of fluorescence production in the presence or absence of RoxS *(ΔroxS*). We also introduced these GFP fusions in *ΔroxS* strains complemented with either a WT (RoxS+) or mutant version of RoxS in which three C-residues of CRR3 were mutated to three G’s (RoxS^CRR3/3G^). Indeed, RoxS is predicted to interact with the ribosome binding site of the *ycsA* mRNA primarily by base-pairing between four C residues of CRR3 and four G-residues of the *ycsA* SD sequence (Fig. 4A). This experiment was done in LB because the *ycsA* mRNA was shown to be much more expressed in these conditions in a previous study by Nicolas et al. (17) (Fig. 4; Fig. S5). The rate of fluorescence accumulation during exponential phase increased approximately 1.8 and in a strain deleted for RoxS (*ΔroxS*) compared to WT, and 1.7-fold in the strain complemented with the RoxS^CRR3/3G^ mutant compared to RoxS+, confirming the translational regulation of *ycsA* expression by RoxS. We also determined the effect of the *ΔroxS* deletion on the stability of the *ycsA* mRNA in LB. Similar to the translational effect, the *ycsA* mRNA half-life increased 2.2-fold in a *ΔroxS* strain compared to the WT strain (4.9 min in the WT strain versus 10.7 min in the *ΔroxS* mutant; Fig. 3D). Off note, the level of *ycsA* mRNA was lower at *t* = 0 in the *ΔroxS* strain suggesting an additional transcriptional effect of RoxS on this mRNA.

Although the RoxS^CRR3/3G^ mutant sRNA was unable to repress the *ycsA*-GFP fusion as anticipated, the steady state levels of this mutant RNA were lower than the WT RoxS, suggesting that it is less stable (Fig. S5C). It was therefore difficult to conclude whether the lack of regulation in the RoxS^CRR3/3G^ mutant was due to impaired base-pairing or to the lower levels of the sRNA. To directly confirm the interaction between the two RNAs, we performed an electromobility shift assay (EMSA) using an *in vitro*-transcribed fragment of *ycsA* containing the SD sequence and either RoxS^CRR3/3G^ or a similar mutant in CRR1 (RoxS^CRR1/3G^) as a control (Fig. 4C). The *ycsA* RNA formed a shifted species with WT RoxS and with the RoxS^CRR1/3G^ mutant, but failed form a complex with the RoxS^CRR3/3G^ mutant, suggesting that the two RNAs indeed interact directly. The introduction of the compensatory mutation in *ycsA* mRNA, where three G’s of the SD sequence were replaced by three C’s, abolished the interaction with WT RoxS and RoxS^CRR1/3G^, but partially restored the interaction with the RoxS^CRR3/3G^ mutant, confirming the interaction site prediction.

### Genes whose expression is upregulated by RoxS.

Only one potential positively regulated RoxS target, *ytsJ*, was identified by both RNAseq and pulsed SILAC (Fig. 1). YtsJ is thought to be the primary malate dehydrogenase of four such enzymes identified in B. subtilis (18). In a previous study we showed that RoxS expression is induced by addition of malate to the growth medium and that RoxS positively regulates the expression of a second malate transporter (YflS) by protecting its mRNA from degradation by RNase J1 (11). Of note, the YflS protein was not detected by pulsed SILAC in this study, due to its localization in the membrane (see next section).

**FIG 5:**
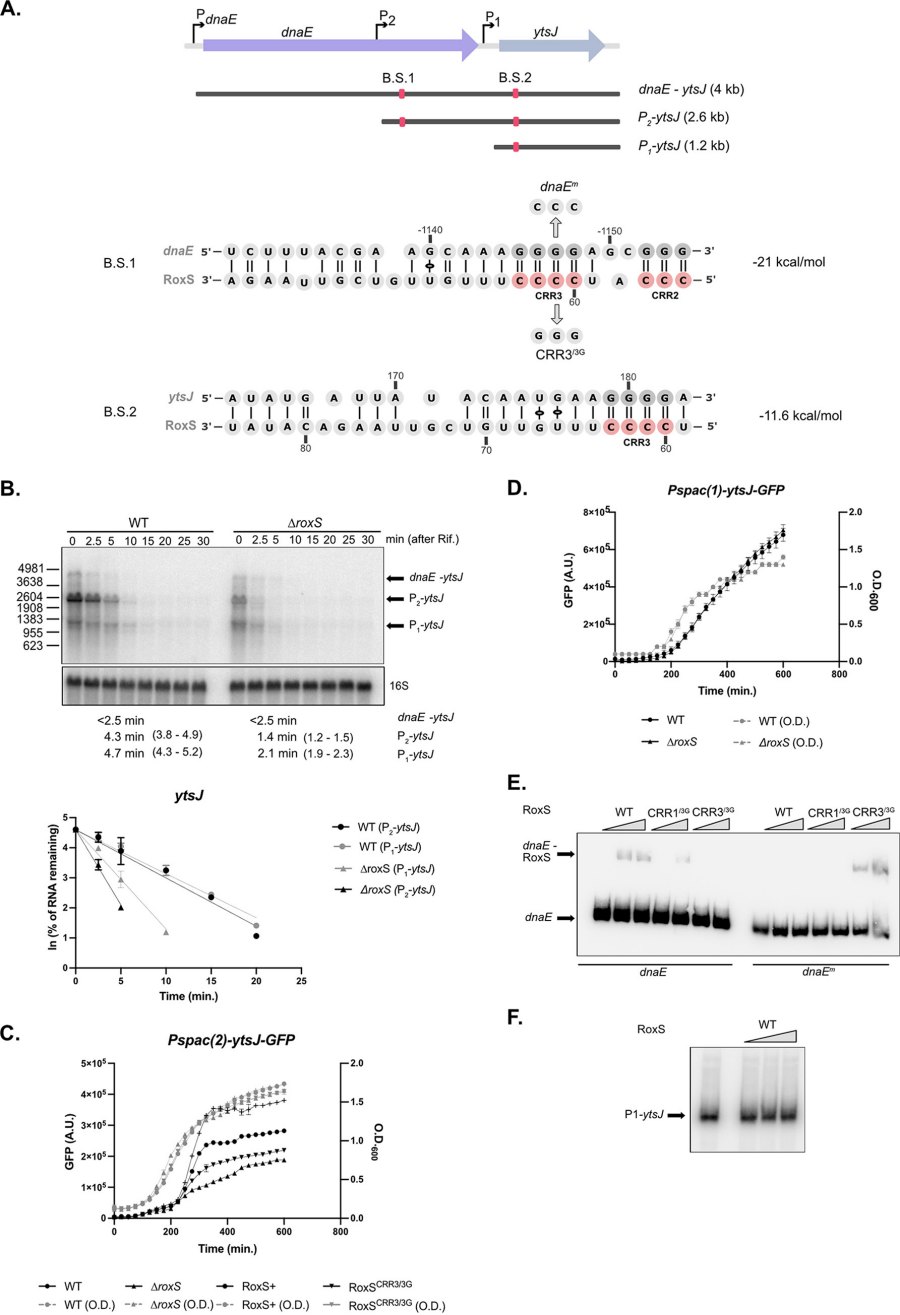
RoxS regulates expression of the P2*-ytsJ* transcript. (A) Genomic environment of *ytsJ* gene. The three promoters mapped in this region, PdnaE, P2, and P1 are shown as black arrows. Red squares indicate the locations of the predicted RoxS binding sites 1 (B.S.1) and 2 (B.S.2), with the interacting sequences shown. Variants of RoxS and *ytsJ* mRNA used for EMSA experiment (E) are also indicated. (B) Northern blot of total RNA from WT and *ΔroxS* mutant strain isolated at different times after addition of rifampicin. Cells were grown in 2xTY + malate (0.5%) and probed for *ytsJ* mRNA. The blot was re-probed for 16S rRNA as a loading control. Graphs and calculated half-lives from two independent experiments are shown beneath the autoradiographs. 95% confidence intervals (CI) are indicated between brackets. (C) and (D) Fluorescence (black lines) produced by the Pspac(2)-*ytsJ-*GFP and Pspac(1)-*ytsJ-*GFP translational fusions, respectively, during growth (gray dashed lines) in LB + malate (0.5%) of WT, *ΔroxS*, RoxS+, and RoxS^CRR3/3G^ cells. For each strain, the rate of fluorescence accumulation was calculated during exponential phase of growth (Fig. S5B) (E) EMSA experiments between WT and mutant variants of RoxS sRNA (RoxS^CRR1/3G^: residues 28 to 30, RoxS^CRR3/3G^: residues 60 to 62) (5 and 10 pmol) and the P2*-ytsJ* mRNA transcript (*dnaE*) (5 pmol) or its variant (*dnaE^m^*) (F) EMSA experiments between the RoxS sRNA and P1-*ytsJ* mRNA.

Previous studies have suggested that *ytsJ* is expressed from three different promoters, located in front of the upstream gene *dnaE* (*PdnaE*), in the middle of the *dnaE* ORF (*P2*) (17) or immediately upstream of *ytsJ* (*P1*) (Fig. 5A) (18). We confirmed the 5′ ends of the *P2*-*ytsJ* and P1-*ytsJ* transcripts within the *dnaE* ORF by primer extension (Fig. S6) and they fit well with the promoter sequences previously proposed (17, 18). Interestingly, a binding site for RoxS was predicted by IntaRNA within the *dnaE* ORF, approximately 190 nt downstream of the *P2* promoter (Fig. 5A). A second RoxS binding site was also predicted within the *ytsJ* coding sequence (CDS), with a lower energy score.

We first measured the half-lives of the various *ytsJ* transcripts in a WT versus a *ΔroxS* strain grown in the same medium as the RNAseq experiment (MD + malate) by Northern blotting using a riboprobe against the first half of the *ytsJ* coding sequence. Surprisingly, none of the three expected *ytsJ* primary transcripts, or an additional species of unknown origin, showed a measurable decrease in half-life (Fig. S7), despite the 1.4-fold increase in mRNA levels found by RNAseq (Table 1). Given that our previous studies have shown that RoxS regulation can depend on growth medium (11, 12), one possible explanation was greater RoxS expression under the control of the arabinose-dependent promoter in the RNAseq experiment compared to that naturally occurring in MD + malate. (9, 10). Indeed, in rich medium (2xTY + malate), we were able to confirm the stabilizing effect of RoxS on *ytsJ* transcripts (Fig. 5B). The half-life of the predominant *P2-ytsJ* (~2.7 kb) transcript was reduced 2-fold in the *ΔroxS* strain compared to WT (5.3 versus <2.5 min). A similar effect was observed with the transcript corresponding in size to *P1-ytsJ* (5.6 versus <2.5 min), while no impact was seen on the bicistronic *dnaE-ytsJ* species (Fig. 5B).

We next tested the effect of RoxS on the translation efficiency of *ytsJ* mRNA, by constructing two GFP fusions, expressed under the control of a Pspac promoter to remove complications of transcriptional effects from the native promoters. These fusions have a transcriptional start close to the P1 (*Pspac(1)-ytsJ-gfp*) or P2 promoter (*Pspac(2)-ytsJ-gfp)*. The experiments were done in LB + malate to reduce background fluorescence from the 2xYT medium. Consistent with the pulsed SILAC data where the YtsJ protein expression was induced 1.5-fold by RoxS, the rate of fluorescence accumulation of the P2-YtsJ-GFP fusion was 3.5-fold lower in the *ΔroxS* strain compared to WT, confirming that RoxS increases YtsJ expression (Fig. 5C; Fig. S5B). However, we were unable to detect an impact of RoxS on the P1-YtsJ-GFP fusion (Fig. 5D). There results confirm that RoxS positively regulates expression of *Pspac(2)-ytsJ-gfp* transcript. To confirm the predicted interaction with CRR3, we measured the rate of fluorescence accumulation in the *ΔroxS* strain complement with either WT (RoxS+) or mutant RoxS (RoxS^CRR3/3G^). As anticipated, the rate of fluorescence accumulation of the P2-YtsJ-GFP in the strain expressing RoxS^CRR3/3G^ was 1.7-fold lower than the WT strain and 2.5-fold lower than the complemented (RoxS+) strain. However, as mentioned previously, the steady state levels of the RoxS^CRR3/3G^ variant are lower than WT RoxS, making it difficult to definitively confirm the interaction with CRR3 in this way (Fig. S5C). To conclusively determine whether RoxS can bind the P2-*ytsJ* transcript, we tested the ability of RoxS to bind to RNA fragments containing the predicted binding sites by gel shift assay *in vitro* (Fig. 5E). As expected, WT RoxS was able to bind to an RNA fragment within the *dnaE* ORF containing the binding site downstream of P2 and this interaction was abolished with the RoxS^CRR3/3G^ mutant. A compensatory mutation in *dnaE* (*dnaE*^m^) restored base-pairing with the RoxS^CRR3/3G^ mutant, confirming their ability to interact *via* the CRR3 region. We were unable to detect binding between RoxS and a fragment within the *ytsJ* ORF, containing the second potential binding site downstream of P1 (Fig. 5F), consistent with the lack of regulation seen with the P1*-ytsJ-gfp* fusion. Together, these results suggest that RoxS directly regulates *ytsJ* expression from the P2 transcript, but not that from P1. The explanation for the effect of RoxS on the half-life of the *P1-ytsJ* transcript is unclear.

**FIG 6:**
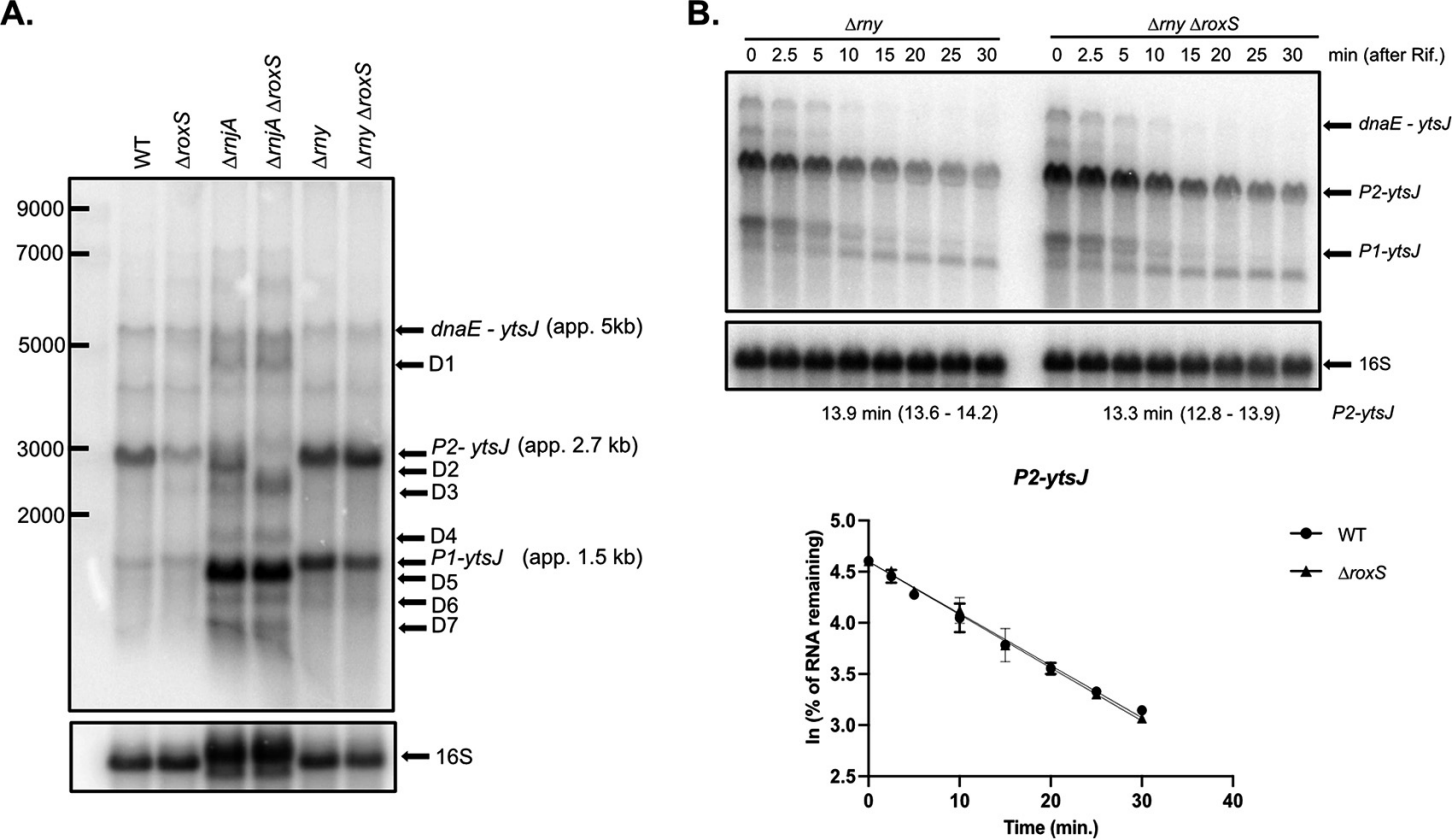
RoxS protects an RNase Y cleavage product of the P2-*ytsJ* transcript from degradation by RNase J. (A) Northern blot showing various transcripts and degradation products of *ytsJ* detected in WT, *ΔroxS*, *ΔrnjA*, *ΔrnjA ΔroxS*, *Δrny*, and *Δrny ΔroxS* strains. The Northern blot was re-probed for 16S rRNA as a loading control (note that a 16S processing intermediate accumulates in the *rnjA* background). (B) Northern blot of total RNA from *Δrny* and *Δrny ΔroxS* mutant strain isolated at different times after addition of rifampicin. Cells were grown in 2XTY + malate (0.5%) and probed for *ytsJ* mRNA. The blot was re-probed for 16S rRNA as a loading control. *ytsJ* (P1) and (P2) indicate *ytsJ* transcript from the P2 and P1 promoter, respectively. Graphs and calculations from two independent experiments are shown beneath the autoradiographs. 95% confidence intervals (CI) are indicated between brackets.

The position of the RoxS binding site far upstream (approximately 1 kb) of the *ytsJ* start codon on the P2 transcript suggested that the primary effect might be on mRNA stability. To determine whether RoxS protects the P2-*ytsJ* mRNA against RNase J1 as observed previously for the upregulated *yflS* mRNA (11), or against RNase Y, one of the main endoribonucleases involved in mRNA degradation in B. subtilis, we analyzed the degradation profile of *ytsJ* mRNAs in *ΔrnjA and Δrny* mutant strains in presence or absence of RoxS (Fig. 6A and B). These experiments were done in rich medium (2XTY) where the P2-*ytsJ* transcript is best detectable. The steady-state levels and stability of P2-*ytsJ* (and indeed all *ytsJ* transcripts) were no longer sensitive to RoxS in the *Δrny* mutant 13.9 min in both strains), suggesting that RoxS cannot stabilize *ytsJ* without prior action of RNase Y (Fig. 6A and B). In the *ΔrnjA* mutant, up to seven different degradation intermediates were detected compared to the WT strain (D1 to D7; Fig. 6A). Two of these were impacted by the deletion of the *roxS* gene; D2 disappeared and was replaced by an intensified D3. This suggests that RoxS protects D2 from degradation by RNase J1. To clarify how RoxS might protect D2 from RNase J1, we performed a primer extension assay in the different RNase mutant strains to identify the 5′ ends of the degradation products (Fig. S6). Because the signal detected from the native *ytsJ* gene was very low, we overexpressed a part of the 5′ UTR of the *P2-ytsJ* transcript to increase the intensity of the bands (Fig. S6). The 5′ end corresponding to D2 was highly intensified in a *ΔrnjA* mutant strain and was absent in strains lacking either RoxS or RNase Y, suggesting that D2 is generated by RNase Y cleavage and protected from RNase J1 degradation by RoxS. Interestingly, this 5′ end is only a few nucleotides (3 to 4) upstream of the RoxS binding site, a compatible location to block the processive activity of RNase J1 (19), similarly to what we previously observed for the *yflS* mRNA (11). The primer extension profile in the region of the second potential RoxS binding site in the *ytsJ* ORF was insensitive to the presence of RoxS in the different RNase mutant strains, reinforcing our conclusion that RoxS does not bind in the CDS of the *ytsJ* mRNA.

### Targets identified only by RNAseq.

A number of potential targets were identified by RNAseq, but not by pulsed SILAC. Most of these mRNAs are predicted to encode membrane proteins that would not have been present in the soluble protein fraction analyzed in the pulsed SILAC experiment, such as the *yfls* mRNA, encoding a malate transporter, which has been previously shown to be protected against the 5′–3′ exoribonuclease J1 by RoxS pairing in B. subtilis (11). Secreted proteins are a second class of proteins absent of the soluble fraction analyzed by pulsed-SILAC. This is the case for the VPR protein encoded by the *vpr* mRNA identified by RNAseq. IntaRNA predicted RoxS binding site(s) in all cases but one (the *cidAB* operon), with energy scores of <-5 kcal/mol (see Materials and Methods). Although the other interactions remain to be validated, it is interesting that almost 50% of the downregulated targets identified by RNAseq have a classical predicted binding site in their SD region.

One upregulated target, *yfiY* mRNA, which encodes a siderophore-binding subunit of an ABC transporter, has a predicted binding site for RoxS at its extreme 5′-end. The position of this binding site is similar to that described for *yflS* mRNA, where RoxS protects from the degradation by the 5′–3′ exoribonuclease J1 (11). These base-pairing predictions reinforce our belief that several of these membrane protein-encoding mRNAs are also potentially direct targets of RoxS (Fig. 1).

## DISCUSSION

This work shows that pulsed SILAC is an efficient alternative to identify direct effects of sRNAs on the translation of target genes, in particular downregulatory effects that would normally be masked by protein stability. It is less cumbersome than ribosome-profiling experiments and has the major advantage of only focusing on newly synthesized proteins, without the background noise of translation occurring under equilibrium conditions. Using this technique, we not only identified most of the known targets of RoxS, but we also identified new potential targets for this sRNA in B. subtilis. We observed a clear enrichment of downregulated genes involved in the TCA cycle, in agreement with the proposed function of RoxS to limit flux through this pathway to restrict NADH production in the presence of favorable carbon sources such as glucose or malate. We also identified the *ytsJ* mRNA as a new positively regulated target of RoxS. This gene encodes one of the four malate hydrogenases in B. subtilis, the only one that uses NADP+ instead of NAD+ as cofactor. The preferential upregulation of an enzyme using NADP+ instead of NAD+ as cofactor fits well with the need for the cell to control NADH production. The enrichment of downregulated genes involved in metabolic pathways directly connected to the TCA cycle such as l-glutamate biosynthesis, citrate metabolism, glycine decarboxylation, and gluconeogenesis also is also consistent with the proposed metabolic role of RoxS (Fig. 2). This study also allowed us to identify *ycsA* mRNA as a direct target of RoxS. This target is repressed approximately 2-fold by RoxS. The fact that *ycsA* mRNA encodes a putative tartrate dehydrogenase that uses NAD+ as cofactor is also in good agreement with the proposed function of RoxS.

In contrast to the *ycsA* mRNA, which has a classical binding site for RoxS in its SD region, several mRNAs that seem to be regulated primarily at their translational level by RoxS, have at least one putative binding site predicted in their coding sequence (CDS) instead of the SD region, which is rather unusual (Fig. S8). A few examples have been described in bacteria where the sRNA binds to the CDS well downstream (>5 codons) of the AUG. Some of these sRNAs impact mRNA stability with a likely indirect affect their translation (5, 20). In other cases, it has been shown that sRNA binding within the CDS can prevent formation of a secondary structure that stimulates translation initiation, lowering the translation efficiency of the target mRNA (21). The presence of sRNA binding sites deep within the CDS raise the question of how base-pairing occurs on an actively translated mRNA. Although we were able to confirm that RoxS has no impact on the mRNA stabilities of two mRNAs from this class of potential targets with putative RoxS binding sites within their ORFs: *yisK* (involved in the control of cell division) and *yjiC* mRNA (encoding a potential macrolide glycosyltransferase), we were unable to see an impact of RoxS on translation of these genes using a GFP-*yjiC* fusion or an antibody raised against YisK protein (data not shown). Furthermore, although the bicistronic *dnaE-ytsJ* and P2-*ytsJ* transcripts contain the RoxS binding site localized in *dnaE*, only the stability of the P2-*ytsJ* mRNA was affected by RoxS (Fig. 5), suggesting that translation of *dnaE* competes with RoxS regulation on the longer mRNA. We therefore cannot rule out that some mRNAs of this class may be off-targets due to the overexpression of RoxS or that they are transitory effects that quickly return to equilibrium following a pulse of RoxS expression. That said, some targets, such as *yukB*, probably merit further investigation because nine potential binding sites for RoxS are predicted within its CDS. One could imagine that transitory slow-downs in translation promoted by sRNA binding throughout the CDS could impact the folding of sub-domains of proteins without a major impact on the protein yield.

Another potential advantage of this type of regulation could be its reversibility. Indeed, removal of the sRNA from its nondegraded targets could allow new rounds of translation on negatively regulated targets or reducing the translation of positively regulated mRNAs but in keeping a basal level of expression. This impact on translation without affecting the pool of the mRNA could be a way to limit the need of new rounds of transcription and quickly adapt to changing environment. A parallel can be drawn to the situation in eukaryotes, where translationally repressed RNAs are stored in P-bodies to be recycled when needed (22).

## MATERIALS AND METHODS

### Strains, plasmids, and oligonucleotides.

Strains and plasmids used in this study are presented Table S1 and oligonucleotides in Table S2.

The *lysA::erm* mutation was transferred by transformation of SSB1002 with chromosomal DNA from the strain BKE23380 (23).

Plasmid pDG1662-PxsA (pl801) is a plasmid containing the arabinose inducible promoter of *xsa* gene, amplified using oligonucleotides CC2250 and CC2251. The PCR fragment was cloned between the BamHI and HindIII sites of pDG1662.

Plasmid pDG1662-PxsA-RoxS (pl805) was made by amplification of RoxS by PCR using oligos CC2285/CC2286 and the resulting fragment cloned between the SpeI and EcoRI restriction sites in pl801.

The pDG148-*ycsA*-GFP (pl862) plasmid was made by PCR amplification of *ycsA* from chromosomal DNA using oligonucleotides CC2546/CC2547 and *gfp* from pHM2-5’hbs-GFP plasmid (pl696) using oligonucleotides CC2548/CC572. The two overlapping PCR fragments containing *ycsA* and *gfp* were assembled in new PCR with oligo pair CC2546/CC572. The *ycsA*-GFP fragment was cloned in pHM2 between the EcoRI and BamHI restriction sites. Due to the low expression after insertion of this fusion at the *amyE* locus, this construction was reamplified with oligo pair CC2611/CC2612 to clone it in pDG148 between the EcoRI and PvuII restriction sites.

To overexpress the part of *dnaE* predicted to base-pair with RoxS, we inserted pHM2-Pspac^con^-*dnaE* at the *amyE* locus. This plasmid was constructed by amplification of a part of *dnaE* by PCR using oligo pair CC2703/CC2705. This fragment was cloned in pHM2-Pspac^con^ between BamHI and SalI restriction sites.

The *ytsJ*-GFP translational fusions were made by amplification by PCR of a DNA fragment starting at the +1 promoter of the P2*-ytsJ* mRNA and covering the first 96 AA of the YtsJ CDS using oligo pair CC2815/CC2822. The 3′ UTR of *ytsJ* with its transcriptional terminator was amplified in a PCR using oligo pair CC2823/CC2816. The GFP fragment was amplified using oligo pair CC2821/CC2824. The three overlapping fragments were amplified in a new PCR using oligo pairs CC2815/CC2816. The resulting product, which is a translational fusion of the GFP after the 96th aa of YtsJ CDS, was cloned in pDG148 between the ClaI and HindIII restriction sites (pl912). Because the level of the GFP was too low to be measurable, we place this fusion under the inducible promoter Pspac of the pDG148-Pspac plasmid. The fusion was reamplified from pl912 using oligo pair CC2984/CC2816. The transcriptional start site of this transcript is close to the +1 of the P2 promoter (Pspac(P2)-*ytsJ*-GFP). We also amplified a shorter version of this fusion to get a transcriptional start site close to the +1 from P1 promoter (Pspac(P1)-*ytsJ*-GFP) using oligo pair CC2704/CC2816. Both fusions, Pspac(P1)-*ytsJ*-GFP and Pspac(P2)-*ytsJ*-GFP were cloned into the HindIII restriction site of the pDG148-Pspac plasmid to give pl935 and pl934, respectively.

Pl982 was made by cloning a mutated version of RoxS in CRR3 region (RoxS^CRR3/3G^) in pDG1662 between the EcoRV and SphI restriction sites. The mutation of RoxS was made by using the oligos pairs CC1377/CC2929 et CC1378/CC2928. The two overlapping fragments were amplified in a new PCR using oligo pairs CC1377/CC1378.

### Medium.

Strains were grown in MD medium (24), 2xYT or LB medium as indicated, all supplemented with 0.5% malate.

### Sample preparation for RNAseq and pulsed SILAC experiments.

Strains CCB1244 and CCB1243 were grown in 50-mL MD modified supplemented with 0.5% malate and a limiting concentration of lysine (85.5 μM) with shaking. Cultures were grown to OD_600_ = 0.5 and split in two cultures of 20 mL. For the pulse-labeling, 1% arabinose and 1.7 mM medium ^2^H_4_-lysine or heavy ^13^C_8_-lysine was added to CCB1244 and CCB1243, respectively, for 15 min before harvesting the cultures (10 mL for RNAseq analysis and 10 mL for pulsed SILAC analysis). Experiments were performed in triplicate.

### RNAseq analysis.

Total RNA was isolated from 10 mL culture (pelleted and frozen) by the glass beads/phenol method described previously (25). RNA samples were treated with RQ DNase Promega (37°C for 20 min) to remove potential contaminating chromosomal DNA. rRNA was removed using the RiboZero kit (Illumina), and rRNA depletion and overall RNA quality was analyzed by Bioanalyser (Agilent). cDNA libraries were prepared using the Smarter Stranded RNA-Seq Kit (Clontech) with adapters for multiplexing, according to the manufacturer’s instructions. cDNA concentration and quality were checked by Bioanalyser (Agilent). The six samples were normalized to 2 nM, multiplexed and denatured at a concentration of 1 nM using 0.1 N NaOH (5 min at room temperature) before dilution to 10 pM and loading on a HiSeq Rapid SE65. Reads were mapped by Bowtie 2 (26). The analysis was performed using the R software, Bioconductor (27) packages including DESeq2 (28, 29) and the PF2tools package (version 1.5.3) developed at PF2 (Institut Pasteur). Normalization and differential analysis were carried out according to the DESeq2 model and package (version 1.20.0).

All RNAseq raw data files can be freely downloaded at:

https://www.ncbi.nlm.nih.gov/geo/query/acc.cgi?acc=GSE229415

### Pulsed-SILAC samples preparation.

Proteins were extracted from 10 mL cultures. Cells were lysed twice with a French press, centrifuged to remove cell debris, and proteins were precipitated with 5 volumes of ice cold acetone. Protein extracts from the pulse-labeling experiments (arabinose-induced RoxS small RNA labeled with heavy ^13^C_8_-lysine and control cultures labeled with medium ^2^H_4_-lysine) were mixed in a 1:1 (wt/wt) ratio and 25 μg of proteins were loaded on a 12% acrylamide gel prior to separation according to their molecular weight by SDS-PAGE. The gel was fixed and stained with Coomassie brilliant blue R250. The gel lanes corresponding to pulsed-SILAC biological triplicates were cut alike into five bands and subjected to a manual in-gel digestion with modified porcine trypsin (Trypsin Gold, Promega). After destaining, bands were subjected to a 30-min reduction step at 56°C using 10 mM dithiotreitol in 50 mM ammonium bicarbonate (AMBIC) prior to a 1-h cysteine alkylation step at room temperature in the dark using 50 mM iodoacetamide in 50 mM AMBIC. After dehydration under vacuum, bands were re-swollen with 250 ng of trypsin in 50 mM AMBIC and proteins were digested overnight at 37°C. Supernatants were kept and peptides present in gel pieces were extracted with 1% (vol/vol) trifluoroacetic acid. Corresponding supernatants were pooled and dried in a vacuum concentrator. Peptide mixtures were solubilized in 25 μL of solvent A (0.1% (vol/vol) formic acid in 3% (vol/vol) acetonitrile) for mass spectrometry analysis.

### Tandem mass spectrometry of SILAC-labeled protein extracts.

Mass spectrometry analyses were performed on a Q-Exactive Plus hybrid quadripole-orbitrap mass spectrometer (Thermo Fisher Scientific, San José, CA, USA) coupled to an Easy 1000 reverse phase nano-flow LC system (Proxeon) using the Easy nano-electrospray ion source (Thermo Fisher Scientific). Each peptide mixture was analyzed in duplicate. Five μL were loaded onto an Acclaim PepMap precolumn (75 μm × 2 cm, 3 μm, 100 Å; Thermo Fisher Scientific) equilibrated in solvent A and separated at a constant flow rate of 250 nL/min on a PepMap RSLC C18 Easy-Spray column (75 μm × 50 cm, 2 μm, 100 Å; Thermo Fisher Scientific) with a 90-min gradient (0% to 20% B solvent [0.1% (vol/vol) formic acid in acetonitrile] in 70 min and 20% to 37% B solvent in 20 min).

Data acquisition was performed in positive and data-dependent modes. Full scan MS spectra (mass range *m/z* 400 to 1,800) were acquired in centroïd with a resolution of 70,000 (at *m/z* 200) and MS/MS spectra were acquired in centroid mode at a resolution of 17,500 (at *m/z* 200). All other parameters were kept as described in (30)

### Data processing and SILAC quantification.

Raw data were processed using the MaxQuant software package (http://www.maxquant.org, version 1.5.6.5) (31). Protein identifications and target decoy searches were performed using the Andromeda search engine and the UniprotKB database restricted to Bacillus subtilis taxonomy (release: 01/2019; 4,260 entries) in combination with the Maxquant contaminant database (number of contaminants: 245). The mass tolerance in MS and MS/MS was set to 10 ppm and 20 mDa, respectively. Methionine oxidation, protein N-terminal acetylation as well as heavy ^13^C_8_-lysine and medium ^2^H_4_-lysine were taken into consideration as variable modifications whereas cysteine carbamidomethylation was considered as fixed modification. Trypsin was selected as the cutting enzyme and a maximum of two missed cleavages were allowed. Proteins were validated if at least two unique peptides with a peptide FDR < 0.01 were identified. The setting “Match between runs” was also taken into consideration to increase the number of identified peptides. For quantification, we only used unique peptides with a minimum ratio count ≥ 1. Protein ratios were calculated as the median of all peptide ratios for the protein of interest and a normalization step was also included in the quantification process.

### Statistical analysis of the pulsed-SILAC data set.

Statistical analysis was done using Perseus software (https://maxquant.org/perseus, version 1.6.0.7) on normalized protein ratios. Proteins belonging to contaminant and decoy databases were filtered. For each biological replicate, the median ratios (M/L and H/L) of the two injected replicates were determined. Then, a Student's *t* test (threshold *P*-value < 0.05) was applied to detect statistically relevant variations between these two ratios. Moreover, we considered only (H/L)/(M/L) ratios having a fold change >1.4 for upregulated gene and <0.7 for downregulated gene to validate newly synthetized proteins that are specific to the arabinose-induced RoxS sRNA condition.

### IntaRNA prediction.

The following parameters have been used for the intaRNA prediction. The output parameters were defined as follows: number of interactions per RNA pair: 5; suboptimal interaction overlap: can overlap in target; max absolute energy of an interaction: 0, max delta energy above mfe of an interaction: 100, no lonely base pairs and no GU helix ends. The seed parameters were: minimum of base pairs in seed: 7. The folding parameters were: temperature for energy computation: 37°C; folding window size: 150; max. base-pair distance: 100; folding window size: 150; base-pair distance 100; energy parameter set: Turner model 2004. For each prediction, the target sequence encompasses the proximal 5′ and 3′ end of each mRNA determined in Nicolas et al.(17). When the putative target gene is the only gene found to be regulated in an operon, for example ClpQ, 100 nt were added to the 5′ and 3′ ends of the ORF for prediction.

### Gene ontology analysis.

The 83 downregulated genes and the 19 upregulated genes identified by RNAseq and SILAC were used for a gene ontology analysis on the web application “Comparative Go” (14). A hypergeometric test was used to calculate under-represented or enriched biological functions in these data set with a *P*-value ≤0.05.

### GFP measurement.

Strains were grown overnight in LB medium with 0.5% malate and 5 μg/mL phleomycin. The cells were diluted the following day in the same medium. At mid-exponential phase (O.D._600_ = 0.6-0.8) cells were inoculated at (O.D._600_ = 0.01) on 96-well microplates and the O.D._600_ and GFP measured for 15 h. The GFP arbitrary units were corrected with a control strain (CCB1133) with no GFP and an interrupted *amyE* gene because we observed that the AmyE protein increases fluorescence background during growth.

### Electrophoretic mobility shift assays.

For electrophorectic mobility shift assays (EMSAs), RoxS was transcribed with T7 RNA polymerase *in vitro* from PCR templates amplified using the oligo pair CC1482/CC1833. The RoxS CCC to GGG mutation in CRR1(residues 28–30; RoxS CRR1^/3G^) was transcribed from a PCR template amplified using oligo pair CC2831/1833. The RoxS CCC to GGG mutation in CRR3 (residues 60 to 62; RoxS CRR3^/3G^) was transcribed from PCR templates amplified using oligo pairs CC1482/CC2929 and CC2928/CC1833. The overlapping fragments were then assembled in a new PCR and amplified using CC1482/CC1833.

The *ycsA* and the *ycsA*^m^ RNA were transcribed *in vitro* from PCR templates amplified using the oligo pairs CC2644/CC2642 and CC2987/CC2642, respectively. The *dnaE* RNA was transcribed *in vitro* from a PCR template amplified using the oligo pair CC2645/CC2651. The *dnaE*^m^ template was made by reamplification of overlapping PCR fragments amplified using oligo pairs CC2645/CC3179 and CC3178/CC2651. The reamplification step was done using oligos CC2645/CC2651.

Before addition to EMSAs, each RNA was individually heated for 3 min and cooled to room temperature for 10 min. A 15-μL reaction was prepared by mixing 5 pmol of target RNA with increasing concentrations of RoxS (5 and 10 pmol) in 1xRNA binding buffer (10 mM Tris pH 8; 50 mM NaCl; 50 mM KCl, 10 mM MgCl_2_) and incubated at 37°C. After 10 min of incubation, 5 μL of glycerol (stock solution 80%) was added and RNAs were loaded on a 6% non-denaturing polyacrylamide gel (acrylamide:bisacrylamide ratio 37.5:1). Following migration (2.5 h at 230V in cold room), RNA was transferred to a Hybond N+ membrane and hybridized with the radiolabeled probe.

### RNA isolation and Northern blot.

sRNA was isolated from mid-log phase B. subtilis cells growing in 2YT, LB, or MD medium either by the glass beads/phenol method described in (25) or by the RNAsnap method described in (32). Typically, 5 μg RNA was run on 1% agarose or 5% acrylamide gels and transferred to hybond-N membranes (Cytiva) in 0.5X TBE Buffer (100V for 4 h at 4°C). Hybridization was performed using 5′-labeled oligonucleotides or riboprobe labeled with P^32^-UTP using Ultra-Hyb (Ambion) hybridization buffer at 42°C for a minimum of 4 h. Membranes were washed twice in 2 × SSC 0.1% SDS (once rapidly at room temperature [RT] and once for 10 min at 42°C) and then three times for 10 min in 0.2 × SSC 0.1% SDS at RT. Oligonucleotides used are shown in Table S3.

### Data availability.

All LC-MS raw data files as well as Maxquant and Perseus result files have been deposited to the MassIVE repository with the data set identifier MSV000091084 and can be freely downloaded at: https://massive.ucsd.edu/ProteoSAFe/dataset.jsp?accession=MSV000091084.
