# Supplementary figures and images for: New RoxS sRNA Targets Identified in Bacillus subtilis by Pulsed SILAC
Source: Microbiol Spectr. 2023 Jun 20;11(4):e00471-23. doi: 10.1128/spectrum.00471-23 (PMC10433868; doi:10.1128/spectrum.00471-23)

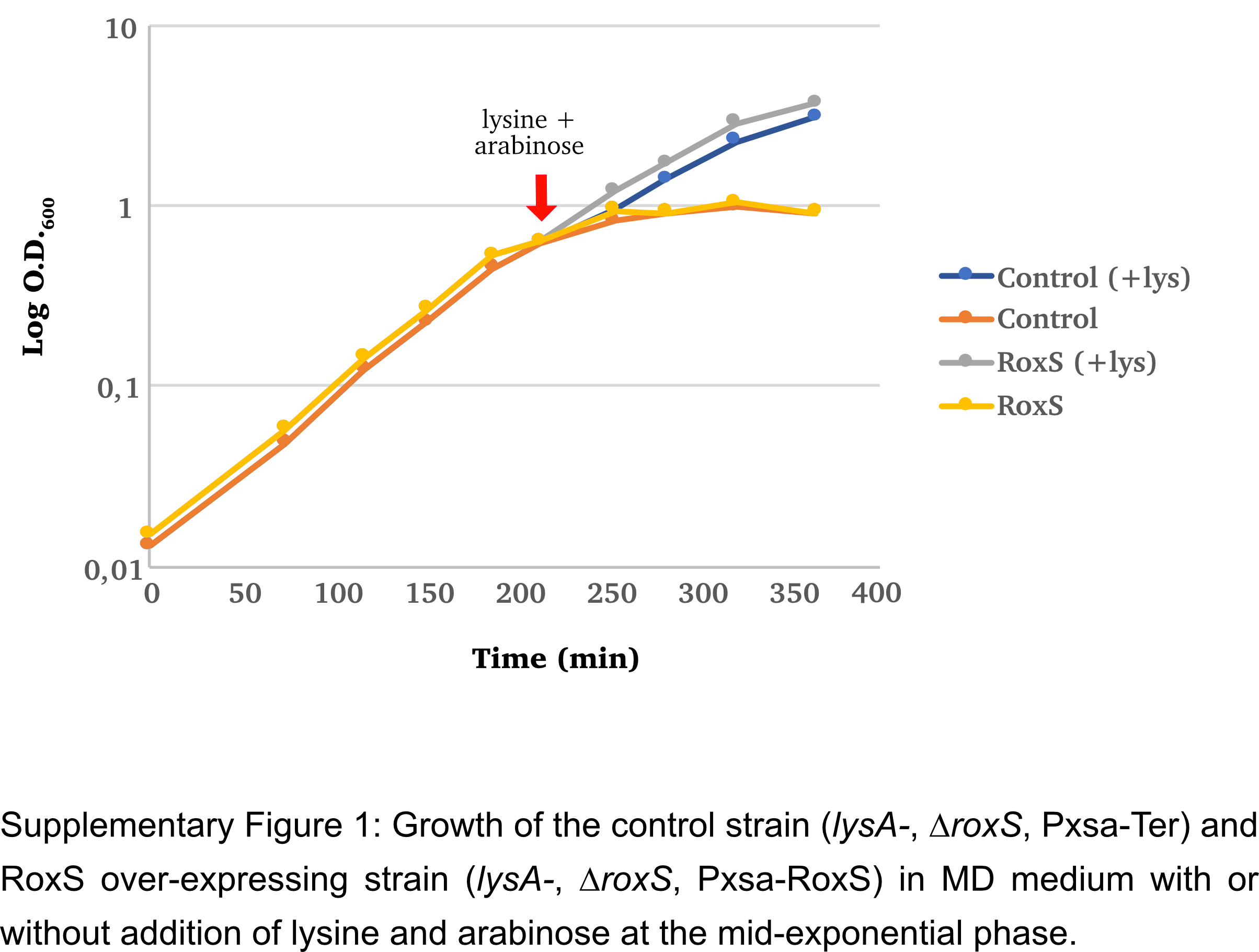

Supplement: Supplemental file 1 — Fig. S1. Download spectrum.00471-23-s0001.tif, TIF file, 0.4 MB [file spectrum.00471-23-s0001.tif]

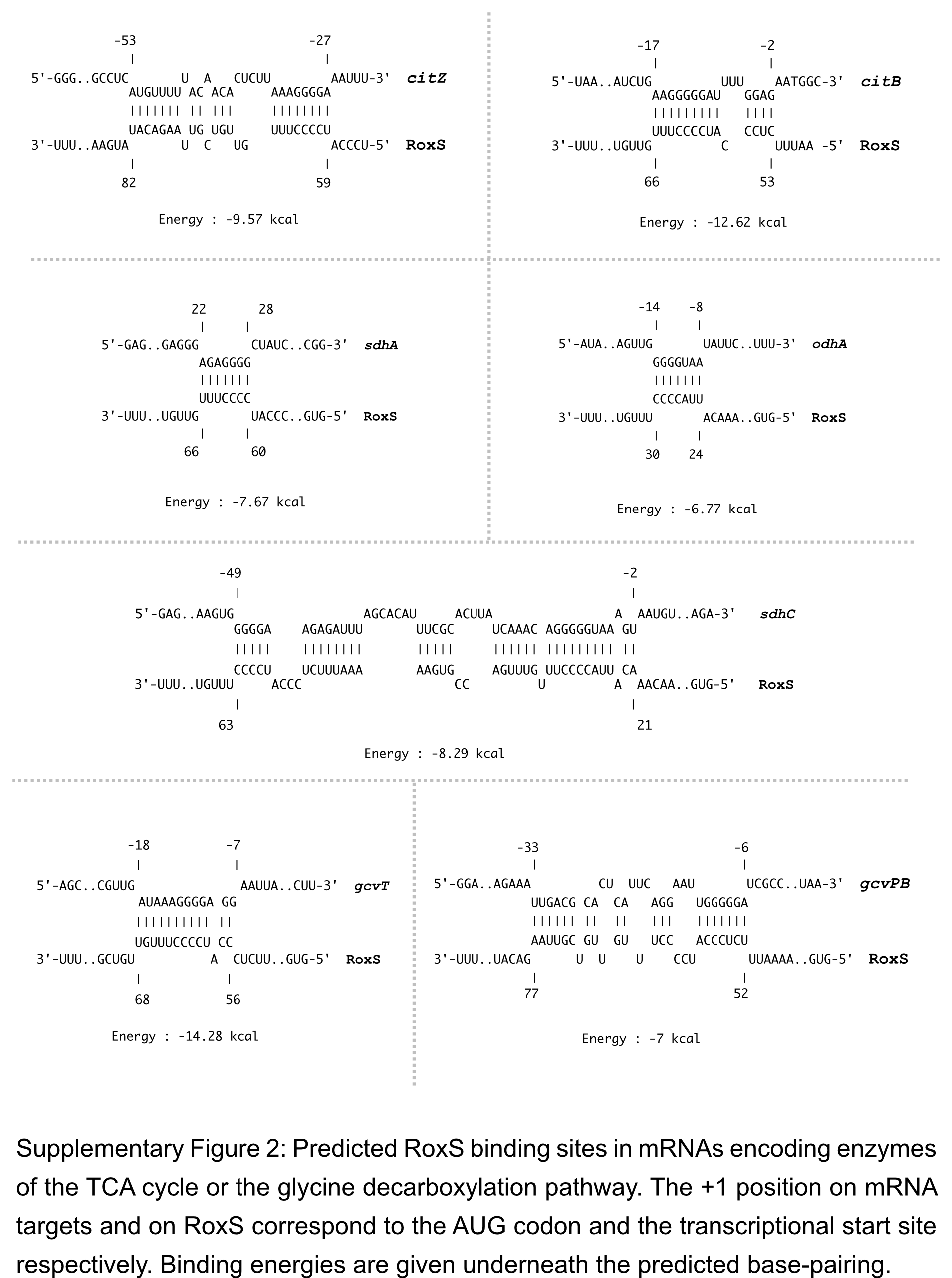

Supplement: Supplemental file 2 — Fig. S2. Download spectrum.00471-23-s0002.tif, TIF file, 0.7 MB [file spectrum.00471-23-s0002.tif]

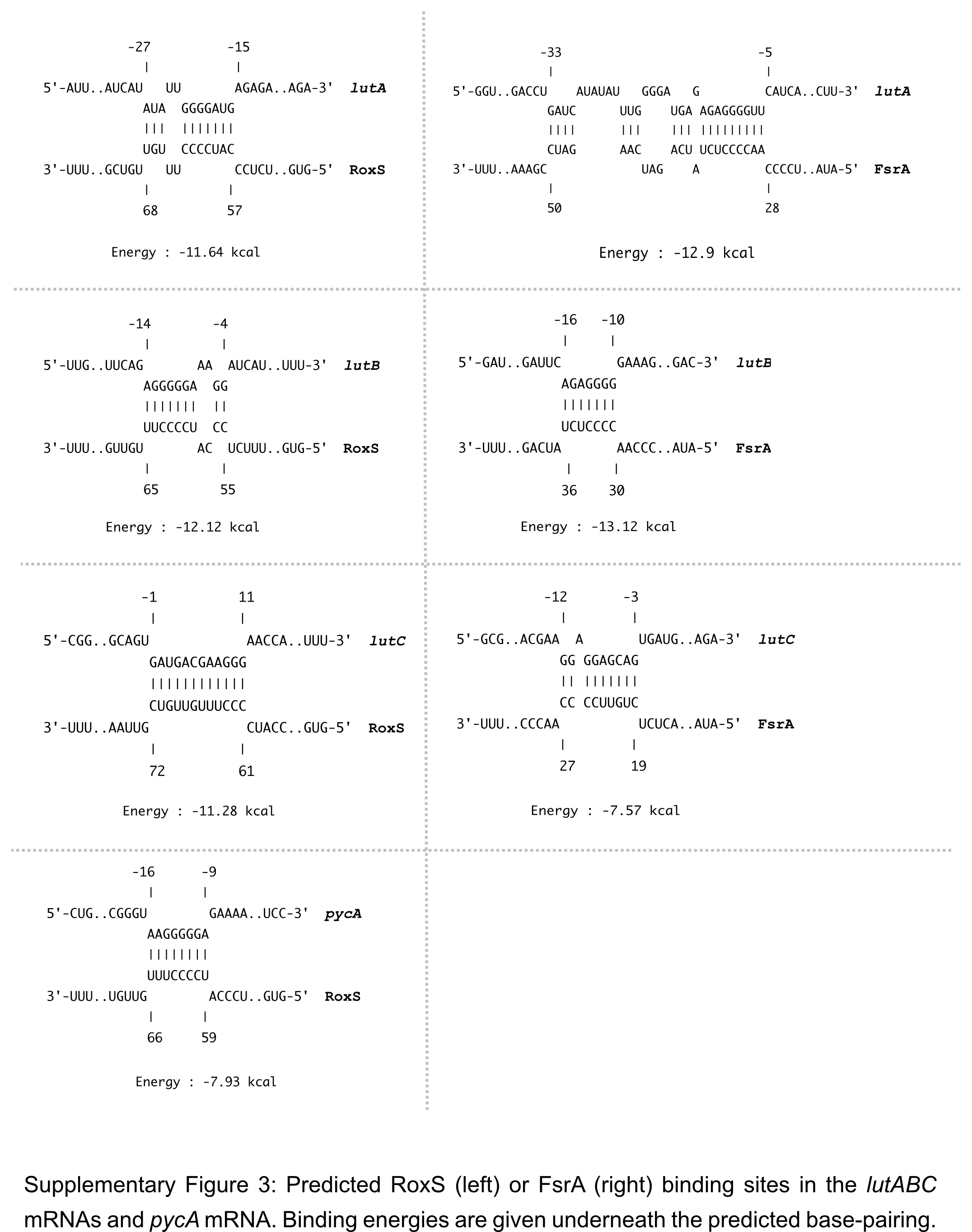

Supplement: Supplemental file 3 — Fig. S3. Download spectrum.00471-23-s0003.tif, TIF file, 0.6 MB [file spectrum.00471-23-s0003.tif]

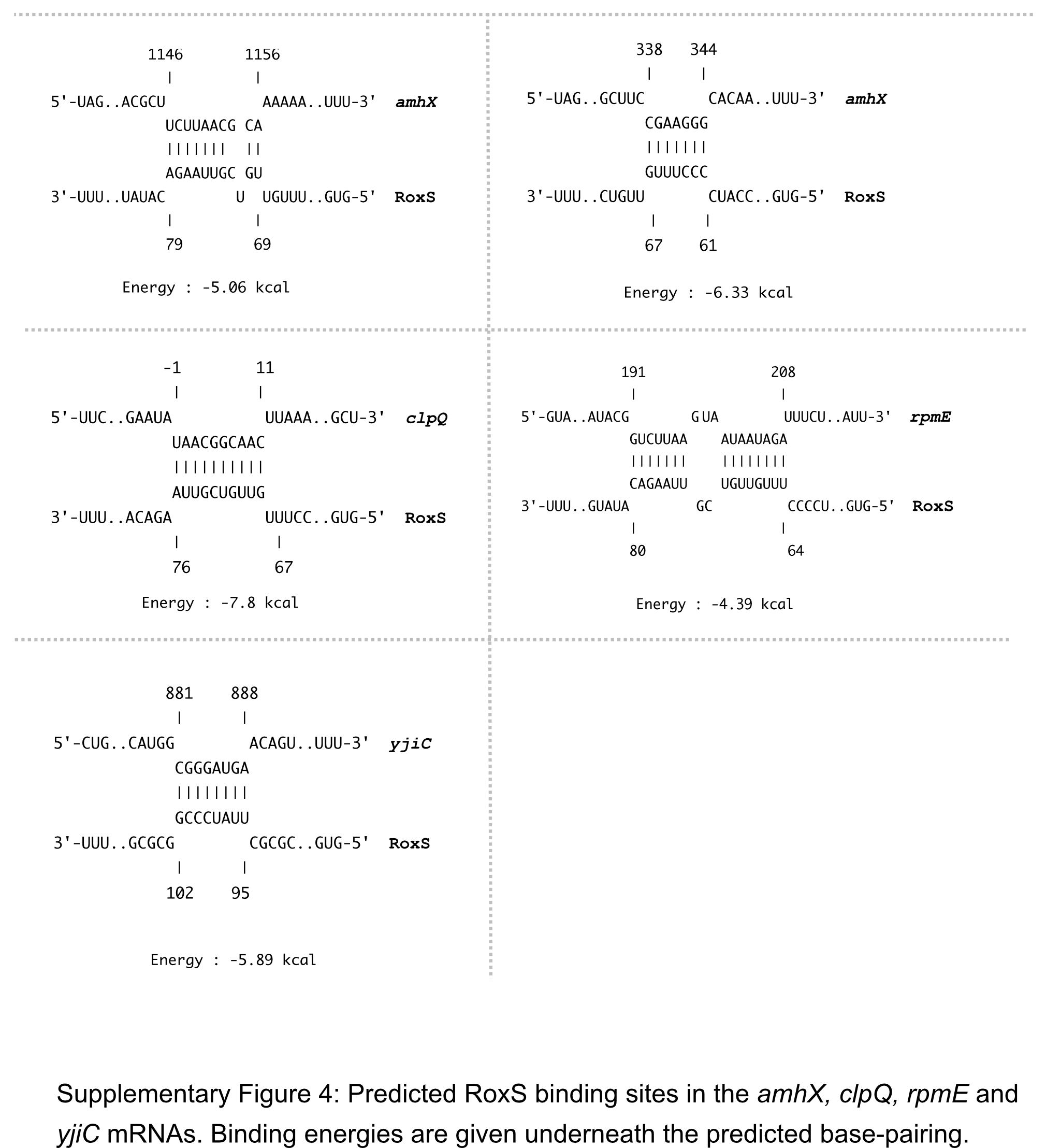

Supplement: Supplemental file 4 — Fig. S4. Download spectrum.00471-23-s0004.tif, TIF file, 0.5 MB [file spectrum.00471-23-s0004.tif]

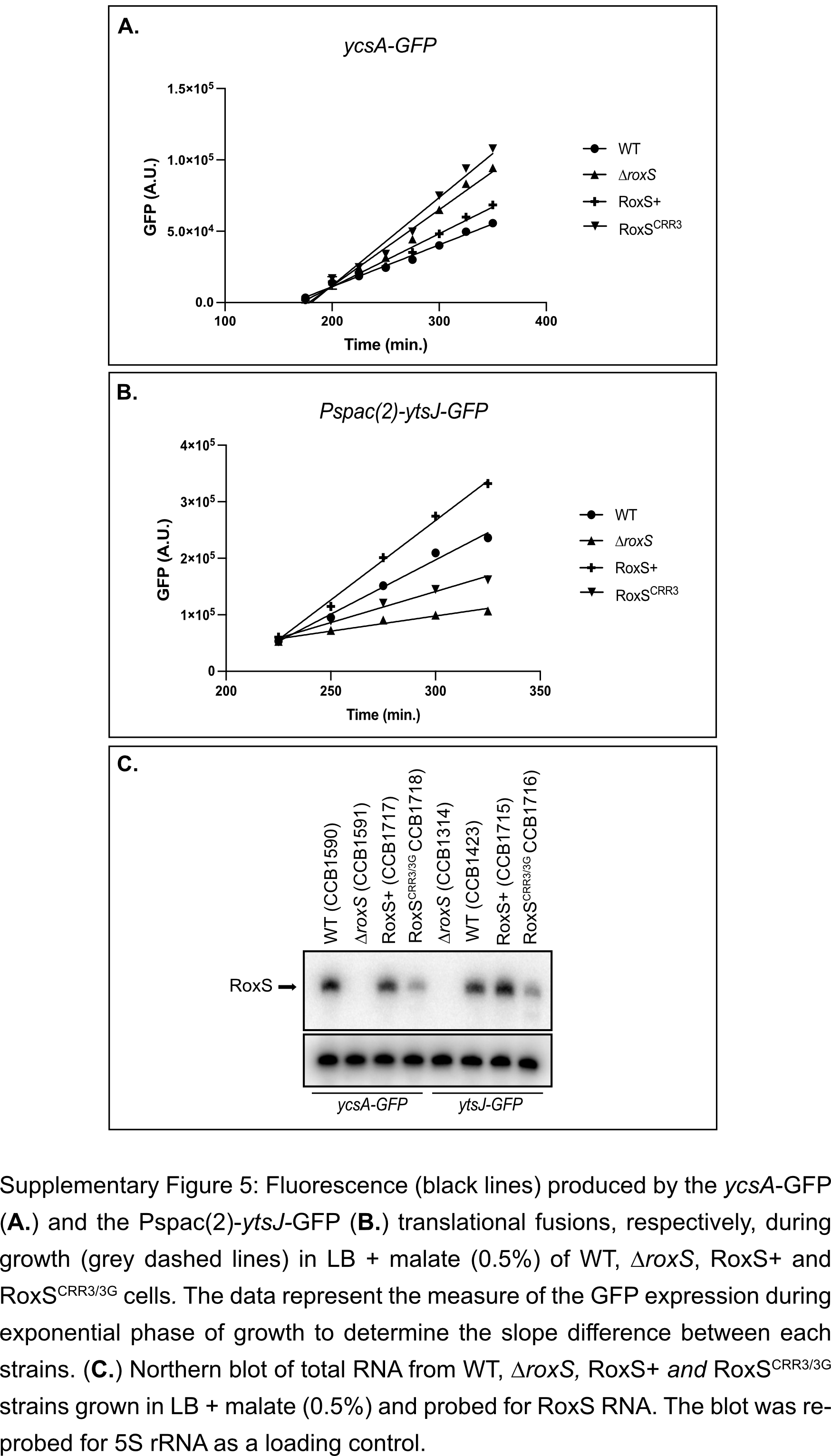

Supplement: Supplemental file 5 — Fig. S5. Download spectrum.00471-23-s0005.tif, TIF file, 0.9 MB [file spectrum.00471-23-s0005.tif]

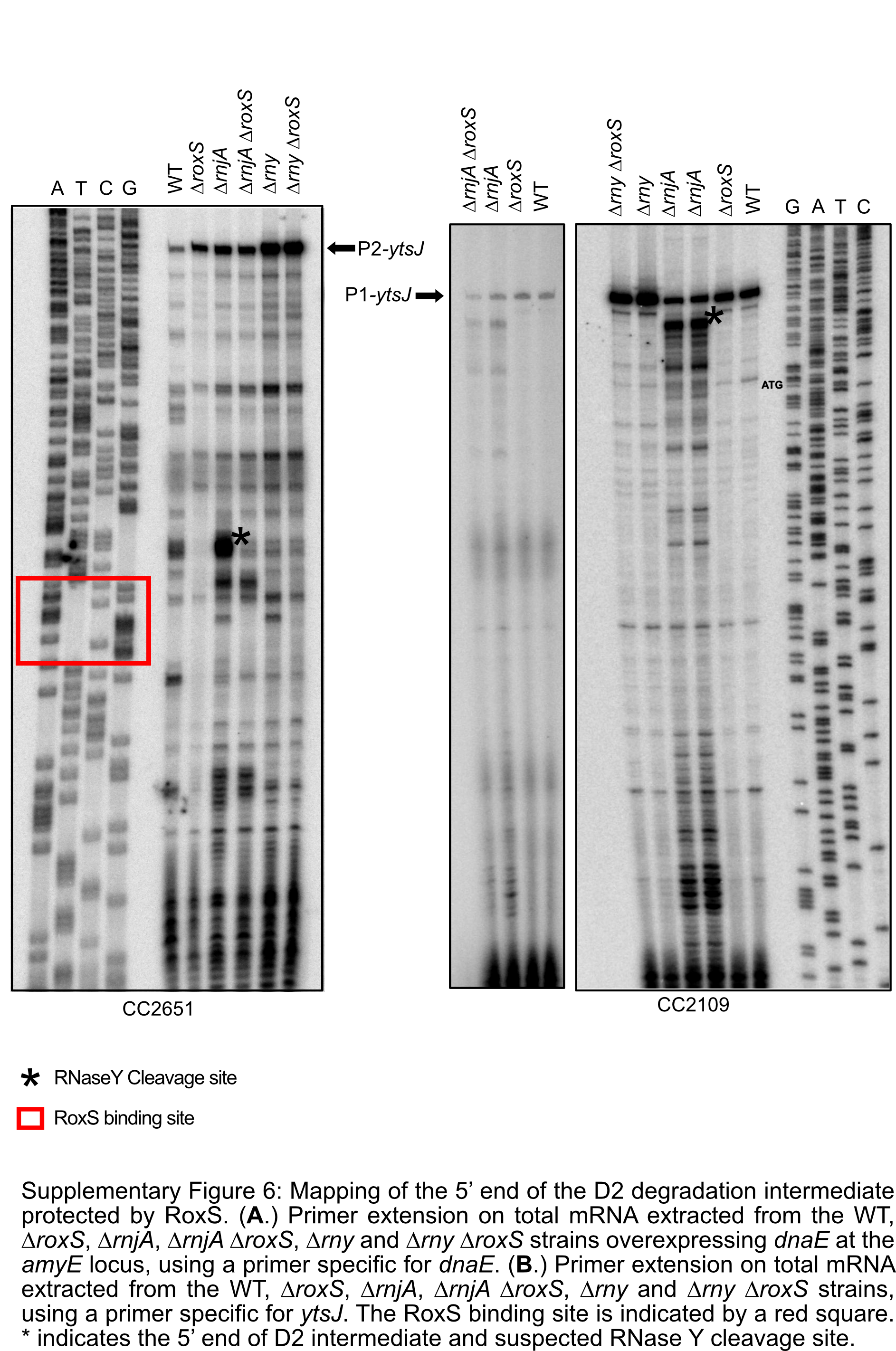

Supplement: Supplemental file 6 — Fig. S6. Download spectrum.00471-23-s0006.tif, TIF file, 2.8 MB [file spectrum.00471-23-s0006.tif]

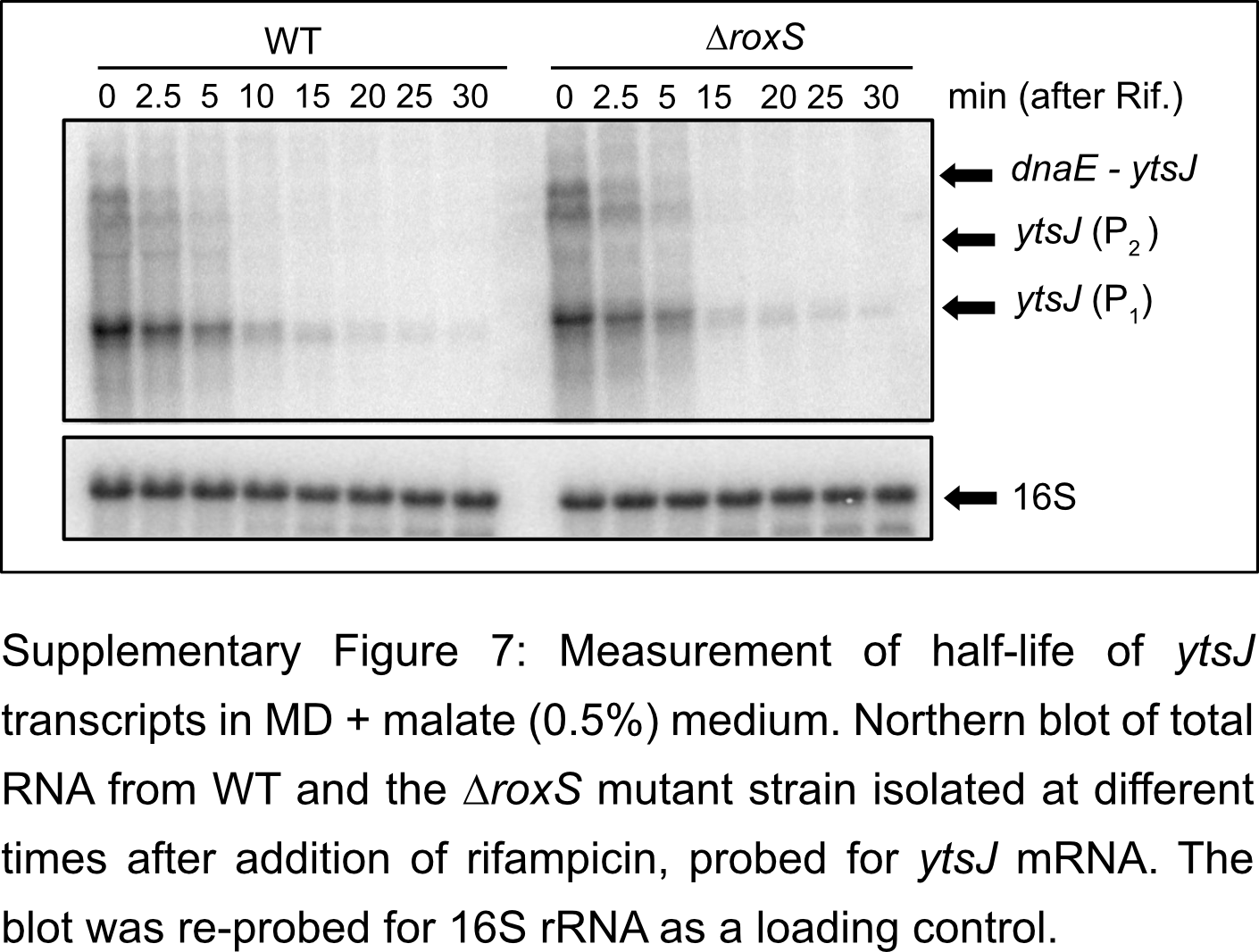

Supplement: Supplemental file 7 — Fig. S7. Download spectrum.00471-23-s0007.tif, TIF file, 0.6 MB [file spectrum.00471-23-s0007.tif]

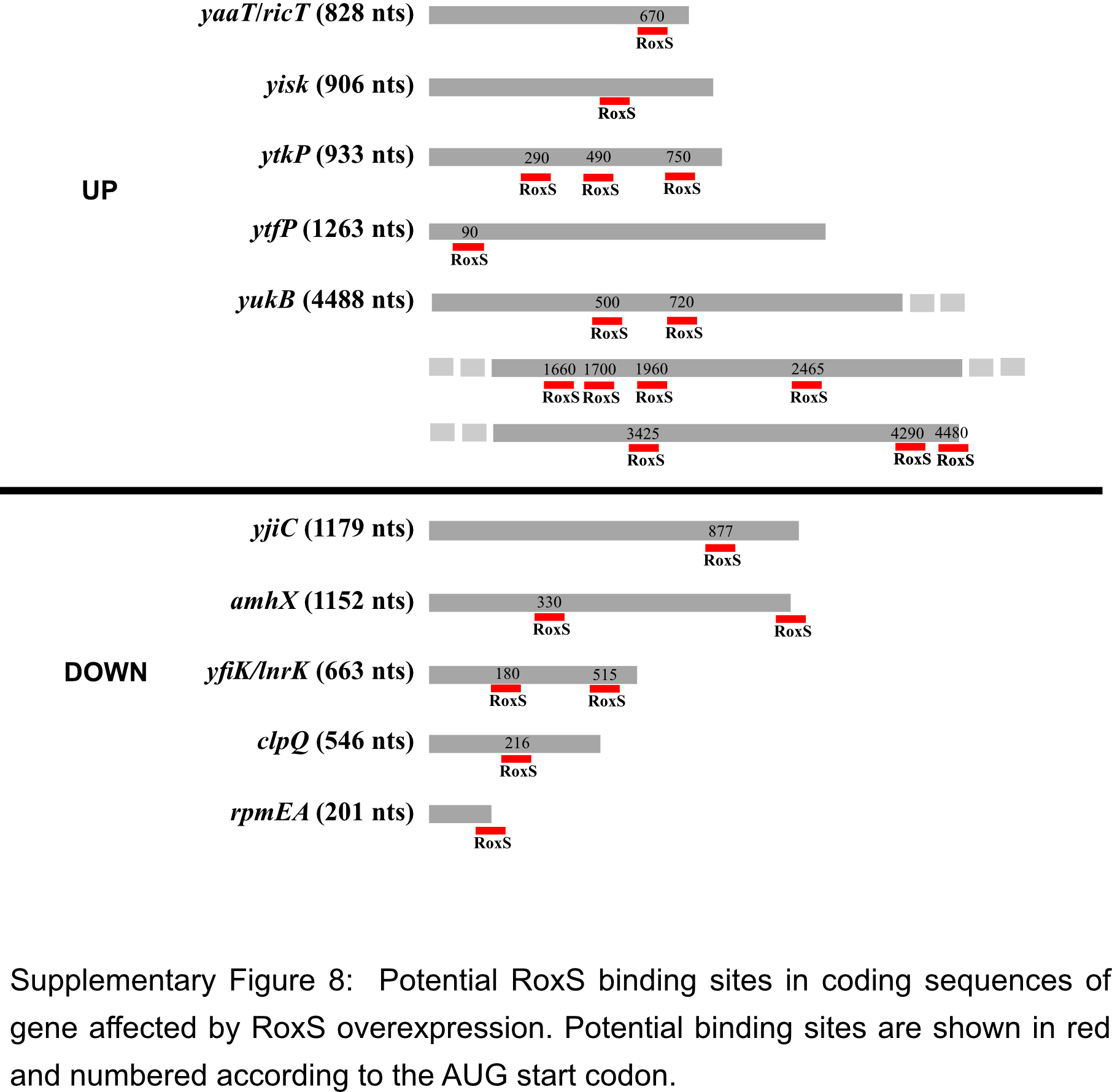

Supplement: Supplemental file 8 — Fig. S8. Download spectrum.00471-23-s0008.tif, TIF file, 0.4 MB [file spectrum.00471-23-s0008.tif]
