# Supplementary material for: New RoxS sRNA Targets Identified in Bacillus subtilis by Pulsed SILAC
Source: Microbiol Spectr. 2023 Jun 20;11(4):e00471-23. doi: 10.1128/spectrum.00471-23 (PMC10433868; doi:10.1128/spectrum.00471-23)
Supplement: Supplemental file 9 — Table S1. Download spectrum.00471-23-s0009.pdf, PDF file, 0.1 MB [file spectrum.00471-23-s0009.pdf]

Strains

Name	Genotype	References
SSB1002	W168 *trpC2*	
CCB441	*rny::spc*	Durand et *al.* 2012
CCB448	*rnjA::kan*	Durand et *al.* 2012
CCB485	*roxS::kan*	Durand et *al.* 2015
CCB558	*roxS::kan rny::spc*	Durand et *al.* 2015
CCB559	*roxS::kan rnjA::kan*	Durand et *al.* 2015
CCB1243	*lysA::ery amyE::pDG1662-Pxsa-RoxS roxS::kan*	This study
CCB1244	*lysA::ery amyE::pDG1662-Pxsa roxS::kan*	This study
CCB1314	*roxS::kan amyE* ::pDG1662-Pxsa pDG148-*ycsA* -GFP	This study
CCB1423	*amyE* ::pDG1662-PxsA pDG148-*ycsA* -GFP	This study
CCB1403	*amyE* :pHM2-Pspac(con)-*dnaE*	This study
CCB1404	*roxS::kan amyE* :pHM2-Pspac(con)-*dnaE*	This study
CCB1411	*rnjA::spc amyE* :pHM2-Pspac(con)-*dnaE*	This study
CCB1412	*roxS::kan rnjA::spc amyE* :pHM2-Pspac(con)-*dnaE*	This study
CCB1413	*rny::spc amyE* :pHM2-Pspac(con)-*dnaE*	This study
CCB1414	*roxS::kan rny::spc amyE* :pHM2-Pspac(con)-*dnaE*	This study
CCB1590	*amyE::* pDG1662-PxsA pDG148-Pspac(P2)-*ytsJ* -GFP	This study
CCB1591	*roxS::kan amyE::* pDG1662-PxsA pDG148-Pspac(P2)-*ytsJ* -GFP	This study
CCB1592	*amyE::* pDG1662-PxsA pDG148-Pspac(P1)-*ytsJ* -GFP	This study
CCB1593	*roxS::kan amyE::* pDG1662-PxsA pDG148-Pspac(P1)-*ytsJ* -GFP	This study
CCB1709	*roxS::kan amyE::pDG1662-RoxSCRR3(3G)*	This study
CCB1715	*roxS::kan amyE::pDG1662-RoxS pDG148-Pspac-ycsA-GFP*	This study
CCB1716	*roxS::kan amyE::pDG1662-RoxSCRR3(3G) pDG148-Pspac-ycsA-GFP*	This study
CCB1717	*roxS::kan amyE::pDG1662-RoxS pDG148-Pspac(P2)-ytsJ-GFP*	This study
CCB1718	*roxS::kan amyE::pDG1662-RoxSCRR3(3G) pDG148-Pspac(P2)-ytsJ-GFP*	This study

Plasmids

	Name	Description
pl805	pDG1662-Pxsa-RoxS	Arabinose inducible promoter of xsa gene cloned in pDG1662. Allows cloning in +1 (SpeI site)
pl801	pDG1662-Pxsa	Integrative plasmid (*amyE*) containing RoxS under control of arabinose dependent promoter Pxsa. Allows tight expression from +1. RoxS cloned between SpeI and EcoR1 restriction sites.
pl862	pDG148-*ycsA* -GFP	Translational fusion of whole YcsA orf with GFP. Overlap PCR: CC2546/2547 and 2548/572. 2nd PCR amplification with CC2546/572. Cloned into the EcoRI/BamHI restriction sites of pHM2. The *ycsA* -GFP fusion from plasmid 854 was recloned in pDG148; oligos CC2611/2612. Cloned in EcoR1/BamHI
pl886	Pspac(con)-*dnaE*	From internal P2 promoter to end of *dnaE* . coordinates 2992494-2991279. Amplified by PCR (CC2703/2744). Cloned into BamHI/SalI
pl934	pDG148-Pspac(P2)-*ytsJ* -GFP	Fusion *ytsJ* -GFP. long 5'UTR (P2). GFP fusion at K(96). cloned into the HindIII restriction site. Under control of the Pspac.
pl935	pDG148-Pspac(P1)-*ytsJ* -GFP	Fusion *ytsJ* -GFP. short 5'UTR (P1). GFP fusion at K(96) cloned into the HindIII restriction site. Under control of the Pspac.
pl982	pDG1662-RoxSCRR3(3G)	Integrative plasmid (*amyE*) containing a mutated version of RoxS in CRR3 region (3C->3G). The fragment was cloned in pDG1662 between EcoRV/SphI sites.

Table S1: Strains and plasmids used in this study.
