# Supplementary material for: New RoxS sRNA Targets Identified in Bacillus subtilis by Pulsed SILAC
Source: Microbiol Spectr. 2023 Jun 20;11(4):e00471-23. doi: 10.1128/spectrum.00471-23 (PMC10433868; doi:10.1128/spectrum.00471-23)
Supplement: Supplemental file 10 — Table S2. Download spectrum.00471-23-s0010.pdf, PDF file, 0.3 MB [file spectrum.00471-23-s0010.pdf]

Oligos

Name	Sequence	Description
		**for Northern blot**
CC2166	CAAAGGGTTTTAGTTCCAGCAGC	Oligo fwd. for synthesis of *citZ* riboprobe
CC2167	GCTCTAATACGACTCACTATAGGGATACCCCACATATGTAAGGGTATCATC	Oligo Rev. with T7 promoter for synthesis of *citZ* riboprobe
CC2577	GCTCTAATACGACTCACTATAGCTCTGTCCTGGCACCTGAAAG	Oligo Rev. with T7 promoter for synthesis of *gcvT* riboprobe
CC2578	CATAACAGCATGAAAATATGAGCG	Oligo fwd. for synthesis of *gcvT* riboprobe
CC3062	GCTCTAATACGACTCACTATAGGCAAGCTGTCCGAGATAGAAATC	Oligo Rev. with T7 promoter for synthesis of *lutB* riboprobe
CC3063	GAGCGGGTATCACAGGGGATTG	Oligo fwd. for synthesis of *lutB* riboprobe
CC2533	GCTCTAATACGACTCACTATAGGGCATTCTGGATGGCGATTTCATC	Oligo Rev. with T7 promoter for synthesis of *ycsA* riboprobe
CC2534	CTTGGAGCACGGCAAAATGATGC	Oligo fwd. for synthesis of *ycsA* riboprobe
CC2826	GCTCTAATACGACTCACTATAGGGCGCCGCTTCAGGGCCGATGTTTCC	Oligo Rev. with T7 promoter for synthesis of *ytsJ* riboprobe
CC2090	GGAGAATGTCATTAAGAGAAGAAG	Oligo fwd. for synthesis of *ytsJ* riboprobe
		**for primer extension**
CC2651	GCGCGGAGGCTGTATCTGACAGGCGCGGAGGCTGTATCTGACAG	Oligo for mapping of of the P2 promoter of *ytsJ* in *dnaE*
CC2109	GCCGATGTTTCCAAGGCCAAGCAC	Oligo for mapping of of the P1 promoter of *ytsJ* in *dnaE*
		**for strain construction**
CC2250	GACGAAGGATCCCATATTTATAAATACATACGTAC	Oligo fwd. to clone the xsa (abf2) promoter inducible with arabinose in pDG1662.
CC2251	GACGAAAAGCTTGTAAGCGCTTTTACTAGTATTATATTATATATGTTC	Oligo rev. to clone the xsa (abf2) promoter inducible with arabinose in pDG1662.
CC2285	GTCGTGACTAGTGTGAAATTGATCACAAACAAAC	Oligo fwd. to clone RoxS under the inductible arabinose promoter (PxsA) in pDG1662-Pxsa (cloning site SpeI).
CC2286	GTCGTGGAATTCCCAAACGTACGTTCCATTTTTAGCAC	Oligo rev. to clone RoxS under the inductible arabinose promoter (PxsA) in pDG1662-Pxsa (cloning site SpeI).
CC2546	ACATGAGAATTCTTAATCAATAACCGACCACCCG	Oligo fwd. to amplify *ycsA* in order to construct the *ycsA*-GFP fusion (cloning site EcorI)
CC2547	GACAACTCCAGTGAAAAGTTCTTCTCCTTTACTGAGCTTTCTTAAGCGCGAACAG	Oligo rev. to amplify *ycsA* in order to construct the *ycsA*-GFP fusion
CC2548	CTGTTCGCGCTTAAGAAAGCTCAGTAAAGGAGAAGAACTTTTCACTGGAGTTGTC	Oligo fwd. to amplify gfp. Complementary to CC2547
CC572	AAGCAAAGGGATCCCCTGTCAGGAAGCCCATGCTC	Oligo rev. In pHM2 to amplify GFP from pHM2-5'hbs-GFP plamid (cloning site BamHI)
CC2611	ACATGAATCGATTTAATCAATAACCGACCACCCG	Oligo fwd. to amplify *ycsA*-GFP construct from pHM2-*ycsA*-GFP to clone it in pDG148 (cloning site EcorI)
CC2612	AACGAAGGTCGACCCTGTCAGGAAGCCCATGCTC	Oligo rev. to amplify *ycsA*-GFP construct from pHM2-*ycsA*-GFP to clone it in pDG148 (cloning site PvuII)
CC2703	ACACAGGATCCACACTGCAAATGAGGTCTACG	Oligo fwd. to clone the dnaE fragment into pHM2-Pspac© (cloning site BamHI)
CC2705	ATATAGGATCCGACAAGGTTGACAGCGCATTC	Oligo rev. to clone the dnaE fragment into pHM2-Pspac© (cloning site SalI)
CC2815	TATAATCGATGGGCGGTCAGCAAAAAGAAAAAAG	Oligo Fwd. to construct the *ytsJ*-GFP fusion (from P2 promoter)
CC2822	CTCCAGTGAAAAGTTCTTCTCCTTTACTTTTGAAAAGAACTGCTTTCCCTTCC	Oligo rev. overlap GFP-*ytsJ* to construct the *ytsJ*-GFP fusion at the 96 aa of *ytsJ* (from P2 promoter).
CC2821	GGAAGGGAAAGCAGTTCTTTTCAAAAGTAAAGGAGAAGAACTTTTCACTGGAG	Oligo fwd. overlap GFP-*ytsJ* to construct the *ytsJ*-GFP fusion at the 96 aa of *ytsJ* (from P2 promoter).
CC2824	GCGCTGCCATTTTTGGAATGAATTAAAATTATTTGTATAGTTCATCCATGCCATGT	Oligo Rev. overlap ytsJ-GFP pour clonage des fusions ytsJ-GFP.
CC2823	ACATGGCATGGATGAACTATACAAATAATTTTAATTCATTCCAAAAATGGCAGCGC	Oligo Fwd. overlap GFP-*ytsJ* pour clonage des fusions ytsJ-GFP. (3'UTR ytsJ)
CC2816	TATAAAGCTTATTCGATGACGTGCTCGGAAAGG	Oligo Rev. to clone the *ytsJ*-GFP fusion into pDG148. (3'UTR ytsJ)
CC2984	ATATAAAGCTTACACTGCAAATGAGGTCTACG	Oligo fwd. to amplify the *ytsJ*-GFP fusion from pDG148-P2-*ytsJ*-GFP and cloned it into the pDG148-Pspac(c). Fusion Pspac(P2)-*ytsJ*-GFP
CC2704	ACACAGGATCCAATTATGTGATCCTGTTTAAATATTCAGATAG	Oligo fwd. to amplify the *ytsJ*-GFP fusion from pDG148-P2-*ytsJ*-GFP and cloned it into the pDG148-Pspac(c). Fusion Pspac(P1)-*ytsJ*-GFP
		**for EMSA**
CC2644	TAATACGACTCACTATAGGCGCTTACGATAACAGGGGAAGG	Oligo fwd. with T7 promoter for synthesis of *ycsA mRNA*
CC2987	TAATACGACTCACTATAGGCGCTTACGATAACAGCCCAAGGAGAATGACGATG	Oligo fwd. with T7 promoter for synthesis of *ycsAm mRNA*
CC2642	CCGCTGTATGAAGCACTTTCTCAGC	Oligo rev. for synthesis of *ycsA and ycsAm* (3G->3C) mRNA
CC2645	GCTCTAATACGACTCACTATAGGACACTGCAAATGAGGTCTAC	Oligo fwd. with T7 promoter for synthesis of *dnaE mRNA*
CC2651	GCGCGGAGGCTGTATCTGACAGGCGCGGAGGCTGTATCTGACAG	Oligo rev. for synthesis of *dnaE and dnaEm* mRNA
CC3178	CAATATCTTTACGAAGCAAAGCCCAGCGGGATTCGTATCC	Oligo fwd. for synthesis of *dnaE mRNA* (3G->3C)
CC3179	GGATACGAATCCCGCTGGGCTTTGCTTCGTAAAGATATTG	Oligo rev. for synthesis of *dnaE mRNA* (3G->3C)
CC1482	GCTCTAATACGACTCACTATAGGTGAAATTGATCACAAACAAACATTAC	Oligo fwd. with T7 promoter for synthesis of RoxS sRNA
CC1833	AAAGAAACCGCGCCGGGATAAG	Oligo rev. for synthesis of RoxS sRNA
CC2831	GCTCTAATACGACTCACTATAGTGAAATTGATCACAAACAAACATTACGGGTTTGTTTGACCGTG	Oligo fwd. with T7 promoter for synthesis of RoxS CRR1 sRNA (3C->3G)
CC2928	GTGAAAAATTTCTCCCATGGGCTTTGTTGTCGTTAAG	Oligo fwd. for synthesis of RoxS CRR3 sRNA (3C->3G)
CC2929	CTTAACGACAACAAAGCCCATGGGAGAAATTTTTCAC	Oligo rev. for synthesis of RoxS CRR3 sRNA (3C->3G)

Table S2: Oligonucleotides used in this study.
